# Glucose transport dependency defines a therapeutic vulnerability in JAK2V617F-driven myeloproliferative neoplasms

**DOI:** 10.1186/s12964-026-03018-4

**Published:** 2026-06-23

**Authors:** Patrick Weiand, Nicolas Chatain, Marcelo A. Szymanski de Toledo, Julia Moellmann, Tabea Pirker, Siddharth Gupta, Henrike Jacobi, Jelena Lazarevic, Margherita Vieri, Maria Jimena Rodriguez, Steffen Koschmieder, Deniz Nogueira Gezer, Julian Baumeister

**Affiliations:** 1 Department of Hematology, Oncology, Hemostaseology, and Stem Cell Transplantation, Faculty of Medicine, RWTH Aachen University, Aachen, Germany; 2 Center for Integrated Oncology Aachen Bonn Cologne Düsseldorf (CIO ABCD), Aachen, Germany; 3 Department of Internal Medicine I, Cardiology, University Hospital RWTH Aachen, Aachen, Germany

**Keywords:** MPN, JAK2V617F, CALR, MPL, Metabolic reprogramming, HIF-1, GLUT1, GLUT3, Glucose transport

## Abstract

**Background:**

Myeloproliferative neoplasms (MPN) comprise a heterogenous group of hematological malignancies that include polycythemia vera (PV), essential thrombocythemia (ET), and primary myelofibrosis (PMF). Current therapeutic strategies rely on cytoreductive approaches that mitigate disease burden and thromboembolic risk but are not curative. Allogeneic stem cell transplantation remains the only curative option, underscoring the need for novel therapeutic strategies. We previously identified hypoxia–inducible factor 1 (HIF-‍‍1) as a selective vulnerability in JAK2V617F–positive cells, but the underlying metabolic mechanisms remain incompletely defined.

**Methods:**

In vitro studies utilized 32D cells transduced with an empty vector control, *Jak2*WT, or *Jak2*V617F. To evaluate metabolic dependencies, CRISPR-Cas9 was used to generate *Slc2a1* (GLUT1) and *Slc2a3* (GLUT3) knockout clones, which were subsequently characterized via RNA sequencing, extracellular flux analysis, and cellular fitness assays (proliferation, viability, and apoptosis). Pharmacological targeted inhibition of GLUT1/3 was evaluated in human JAK2V617F–mutated post-MPN AML cell lines (SET-2, HEL), primary patient-derived cells and a *Jak2*V617F knock-in mouse model. Combinatorial efficacy was assessed using the JAK1/2 inhibitor ruxolitinib.

**Results:**

JAK2V617F induced HIF-1–dependent metabolic reprogramming, characterized by increased glycolytic flux and oxidative metabolism. Complete abrogation of glucose uptake occurred only upon combined loss of GLUT1 and GLUT3 in *Jak2*V617F cells, revealing functional redundancy between these transporters that sustains enhanced glycolysis. Disruption of glucose uptake selectively induced stress–associated transcriptional programs and replication stress, triggering an S-phase arrest that culminated in apoptosis and impaired viability, specifically in *Jak2*V617F cells.

In vivo, pharmacological inhibition of HIF-1 or GLUT induced a reorganization of erythropoiesis to the spleen but did not ameliorate core disease features. In contrast, in vitro GLUT inhibition robustly reduced cell viability in human SET-2 and HEL cell lines and impaired proliferation, viability, and colony formation in patient-derived PBMCs.

**Conclusions:**

Collectively, these findings establish HIF-1–driven glucose metabolism as a metabolic vulnerability in JAK2V617F-positive MPN. The selective exhaustion of patient–derived clones defines the HIF-1–GLUT1/3 axis as a central, targetable bottleneck. These data provide a mechanistic rationale for further investigation of HIF-1 or GLUT inhibitors, suggesting that targeting this fundamental requirement may help overcome clinical limitations to achieve disease modification and eradicate the malignant clone.

**Supplementary Information:**

The online version contains supplementary material available at 10.1186/s12964-026-03018-4.

## Background

Philadelphia chromosome–negative myeloproliferative neoplasms (MPN) comprise a heterogenous group of diseases driven by clonal hematopoietic stem and progenitor cells (HSPCs) harboring mutually exclusive driver mutations in *JAK2*, *CALR*, or *MPL*. These mutations confer cytokine-independent proliferation and aberrant differentiation of myeloid progenitor cells [1–3]. The most prevalent driver mutation, *JAK2*V617F, is detected in 90% of patients with polycythemia vera (PV) and in 50–60% of patients with essential thrombocythemia (ET) or primary myelofibrosis (PMF) [4]. Due to their proliferative advantage, *JAK2*V617F–positive cells progressively dominate the bone marrow (BM) niche, displacing wild-type (WT) hematopoietic stem cells (HSCs) and resulting in clonal dominance [1, 5].

Current therapeutic strategies for MPN primarily aim to control symptoms and reduce the risk of thromboembolic and cardiovascular complications by normalizing peripheral blood counts. JAK2 inhibitors, including ruxolitinib and fedratinib, as well as interferon-α therapy, can alleviate disease burden and improve clinical outcomes. However, these treatments do not eradicate the malignant clone. At present, allogenic hematopoietic stem cell transplantation remains the only curative option but is restricted to selected patients with advanced myelofibrosis or post-PV/ET myelofibrosis due to its associated morbidity and mortality [6–9]. Consequently, no therapies in routine clinical use specifically target mutated HSPCs, underscoring the need for novel approaches that selectively target vulnerabilities of disease-driving clones [7].

Our group highlighted a potential vulnerability of JAK2V617F–positive HSPCs, recognizing that the constitutive induction and activation of hypoxia signaling in JAK2V617F–positive cells was linked to increased cell survival and proliferation [10]. We were able to show effective and selective reduction in viability and proliferation specifically in JAK*2*V617F–positive cells in murine and patient–derived samples via inhibition of HIF-1 with echinomycin (Ech). Notably, *Jak*2WT cells did not induce HIF-1 stabilization and were mostly unaffected by Ech treatments, indicating unique vulnerability in JAK2V617F–positive cells [10]. These findings were supported by reports which assign a non-essential role to HIF-1α and -2α in healthy HSCs, but an essential role in AML and CML [11–13].

Further transcriptomic and metabolomic profiling of *Jak2*V617F–positive cells by our group and Rao *et al.* revealed a Warburg–like metabolic shift driven by HIF-1 signaling [10, 14, 15]. While metabolic reprogramming supports enhanced proliferation and resistance to apoptosis, it simultaneously introduces targetable metabolic dependencies [15, 16]. Key glycolytic enzymes, including GLUT1, GLUT3, and PFKFB3 were upregulated in *Jak2*V617F–positive cells, which was ameliorated by HIF-1 inhibition or *Hif1α* knockdown (KD) [10]. HIF-1 is already recognized as a central regulator of metabolic rewiring and a potential therapeutic target across multiple hematological malignancies [17–19]. In line with this, targeting of PFKFB3 in JAK2V617F–positive cells induced apoptosis and suppressed proliferation in vitro and in vivo models, with minimal effects on WT HSCs [14]. Recently, selective inhibition of GLUT1 was shown to significantly impair viability and proliferation in murine AML models and patient–derived samples, further supporting the therapeutic potential of exploiting glucose dependency in hematological malignancies [14, 20].

To further define the role of HIF-1 in shaping the metabolic phenotype of MPN and to evaluate its potential as a selective therapeutic target in mutated HSPCs, we investigated HIF-1 inhibition in a murine model and in patient samples harboring driver mutations in either *JAK2*, *CALR*, or *MPL*. This approach extends our previous work, which focused exclusively on HIF-1α in the context of JAK2 mutations [21].

In contrast to other glycolytic enzymes upregulated by HIF-1 signaling in *JAK2*V617F–positive cells such as LDHA or PDK, GLUT1 and GLUT3 are dispensable for the maintenance of WT HSCs, rendering them attractive candidates for *JAK2*V617F–selective targeting [22, 23]. We hypothesize that disruption of glucose uptake phenocopies the pro-apoptotic and anti-proliferative effects previously reported upon HIF-1 inhibition. To test this, we utilized CRISPR-Cas9-mediated knockout (KO) of *Slc2a1* and *Slc2a3*, encoding GLUT1 and GLUT3, in 32D *Jak2*V617F cells. In parallel, we sought to validate both our previous and concurrent findings in a murine *Vav-iCre Jak2*V617F knock-in (KI) model via pharmacological inhibition. To extend our findings from 32D cells and in vivo models to a clinical context, we evaluated the effects of HIF-1 and GLUT1/3 inhibition in primary MPN patient samples.

## Methods

### Cell lines

Murine 32D suspension cells (DSMZ, Braunschweig, Germany) were cultured in RPMI-1640 (Thermo Fisher Scientific, Waltham, USA), supplemented with 10% fetal calf serum (FCS; PAN-Biotech, Aidenbach, Germany and Capricorn Scientific, Ebsdorfergrund, Germany), 1% penicillin/streptomycin (Thermo Fisher Scientific, Waltham, USA) and 10% supernatant of WEHI-3B cells as a source of murine interleukin-3 (IL-3), essential for cytokine–dependent growth of 32D cells. Cells were cultured at 37 °C in a humidified atmosphere containing 5% CO_2_. 32D MPL *Jak2*WT or *Jak2*V617F GLUT1/3 WT or KO cells were cultured in RPMI-1640 supplemented with 10% serum (Capricorn Scientific, Ebsdorfergrund, Germany), murine IL-3 (2 ng/mL, ImmunoTools, Friesoythe, Germany), D-glucose (5 mM, Thermo Fisher Scientific, Waltham, USA) and L-glutamine (1 mM, Thermo Fisher Scientific, Waltham, USA) (low-supplemented RPMI) for MTT, apoptosis, and proliferation assays as well as RT-qPCR and RNAseq. SET2 and HEL cells (DSMZ, Braunschweig, Germany) were cultured in RPMI-1640, supplemented with 10% fetal calf serum (20% for SET2 cells) and 1% penicillin/streptomycin. Cells were cultured at 37 °C in humidified atmosphere containing 5% CO_2_.

### Extracellular flux analysis

Extracellular metabolic flux and plasticity were assessed using Seahorse extracellular flux assays according to the manufacturer’s protocol with an XF96 Seahorse Analyzer (Agilent Technologies, Santa Clara, USA). 2.5 × 10^5^ 32D cells previously incubated for 24 h at 37 °C in the presence of mIL-3 (2 ng/mL, ImmunoTools, Friesoythe, Germany) were washed repeatedly with phosphate-buffered saline (PBS), resuspended in Seahorse XF RPMI1640 (Agilent Technologies, Santa Clara, USA), supplemented to 1 mM L-Glutamine (Thermo Fisher Scientific, Waltham, USA) and seeded onto 96-well Seahorse XF plates. Plates were coated with 50 µg/mL poly-D-lysine (Thermo Fisher Scientific, Waltham, USA) diluted in PBS one day prior. Cells were centrifuged at 200 xg for 1 min without break to promote uniform monolayer adhesion. Following centrifugation, plates were incubated at 37 °C without CO_2_ supplementation for approximately 1 h. Seahorse cartridge ports A to C were loaded with glucose (10 mM, port A), inhibitors of mitochondrial complexes V (oligomycin, 1 µM) or I/III (rotenone/antimycin A, 0.5 µM each) (port B) and 2-deoxy-D-glucose (2-DG) (port C), which were sequentially injected into all wells simultaneously during assay to stimulate, stress and disrupt cellular metabolism. Extracellular flux was measured via extracellular acidification rate (ECAR) and oxygen consumption rate (OCR). Reagents used in injections were included in either XF Glycolytic Stress Assay or XF Glycolytic Rate Assay Kit (Agilent Technologies, Santa Clara, USA).

### CRISPR-Cas9–mediated gene editing

Multiple knockout (KO) clones of the murine 32D cell line previously transduced to express the human thrombopoietin receptor *MPL* and murine *Jak2*WT or *Jak2*V617F were generated using CRISPR-Cas9–mediated genome editing via nucleofection using a 4D-Nucleofector^®^ X Unit in conjunction with a SF Cell Line 4D-Nucleofector^®^ X Kit S (Lonza Bioscience, Basel, Switzerland) in accordance with manufacturers protocol. 32D cells underwent an initial round of single cell dilution to minimize clonal variability and cultured in WEHI-3B-supplemented RPMI-1640 to maximize viability and proliferation 24 h prior to first round of CRISPR. Nucleofection was performed using program CV-137, which is optimized for high transfection efficiency and viability in 32D cells when used with SF kits. GuideRNAs (gRNA) were selected from the pre–designed AltR CRISPR-Cas9 gRNA database (Integrated DNA Technologies, Coralville, USA) for two targets in *GLUT1* exon 3 and 4 and one site in *GLUT3* exon 5 (Table S1).

Following nucleofection, cells were cultured for 24 h in 37 °C, 5% CO_2_. For clonal isolation, 2 × 10² edited bulk cells were seeded into 20 mL of medium and evenly distributed across two 96-well plates to achieve single-cell dilution and screened after approximately one week by brightfield microscopy for single-cell–derived colonies. For downstream validation, approximately 5 × 10⁵ cells were harvested from each clone at multiple time points.

Protein lysates were analyzed by immunoblotting using antibodies listed in Table S4 to assess loss of protein expression. Due to unspecific binding and poor performance of the GLUT3 antibody, KO of *Slc2a3* was primarily validated by Sanger sequencing (Table S2). GLUT1 KO clones identified by immunoblotting were additionally confirmed by sequencing. PCR amplicons spanning the CRISPR target sites were submitted for Sanger sequencing using the Mix2Seq Kit (Eurofins, Ebersberg, Germany). Sequencing chromatograms were aligned and analyzed for insertion–deletion events using CrispID (KU Leuven) and Inference of CRISPR Edits (ICE) analysis software (EditCo Bio, USA) [24, 25].

### Immunoblotting

SDS-Page and Western blot were performed as previously described [26]. Antibodies used are listed in Table S4.

### Glucose uptake assays

32D KO clones of both genetic backgrounds were characterized for their glucose uptake using the bioluminescence-based Glucose Uptake-Glo™ Assay (Promega, Madison, USA). Cells were cultured in RPMI1640 media, with the addition of mIL-3 (2 ng/mL, ImmunoTools, Friesoythe, Germany) to enable proliferation of *Jak2*WT cells. Cells were washed twice with PBS and seeded onto a 96-well white non-transparent plate (Thermo Fisher Scientific, Waltham, USA) in 100 µL PBS. The assay was conducted following manufacturers protocol, incubating cells at RT for at least 1 h after the detection reagent was added. Luminescence was recorded using a Tecan Infinite M200 (Tecan, Männedorf, Switzerland) with an integration time of 1.0 s.

### MTT assays

Metabolic activity of 32D, HEL and SET 2 cells was characterized by seeding cells in analytical triplicates onto a 96-well plate for 72 h in 100 µL of the respective medium detailed in the Cell Lines section. 3 × 10^4^ (32D and SET2) or 2 × 10^4^ (HEL) were seeded per well. After incubation, 10 µL of 3-(4,5-dimethylthiazol-2yl)-2,5-dipheyltetrazolium bromide (5 mg/ mL, MTT) reagent (Sigma-Aldrich, St. Louis, USA) were added to each well using a multichannel pipette, and plates were incubated in the dark at room temperature for 4 h. In case of SET2 cells, plate was incubated at 37 °C, 5% CO_2_ for 4 h. Subsequently, 100 µL of isopropanol-HCL (490 mL isopropanol + 10 mL HCL [2 M]) were added to each well and thoroughly mixed by repeated pipetting. Absorbance was measured using a Multiskan FC Microplate Photometer (Thermo Fisher Scientific, Waltham, USA) at 550 nm. To quantify the interaction between Glutor and ruxolitinib, the Coefficient of Drug Interaction (CDI) was calculated using the formula CDI = AB / (A × B), where AB represents the relative metabolic viability of the combination treatment, and A and B represent the relative viabilities of the respective single-agent treatments.

### Proliferation assays

Proliferation rates of 32D GLUT1/3 KO cells were assessed by seeding 1 × 10^5^ cells of all groups and genetic backgrounds into 6-well plates in 3 mL of low-supplemented RPMI-1640. Every 24 h over a total duration of 72 h, cells were gently resuspended by pipetting, and 10 µL samples were collected to determine cell counts and viability using a CASY cell counter (OMNI Life Science, Bremen, Germany).

### Apoptosis assays

32D GLUT1/3 KO clone cells of all groups and genetic backgrounds were seeded on a 6-well plate 3 mL low-supplemented RPMI for 48 h (3 × 10^5^ initial cells) or 72 h (1 × 10^5^ initial cells). Apoptosis staining was performed according to the manufacturers protocol of Pacific Blue™ Annexin V Apoptosis Detection Kit with 7-AAD (BioLegend, San Diego, USA). Samples were analyzed in a Gallios flow cytometer (Beckmann Coulter, Brea, USA). Flow cytometry data was analyzed using FlowJo v10.10.0 (BD, East Rutherford, USA).

### RNA sequencing

10^6^ cells of GLUT1/3 WT and GLUT1/3 double KO (dKO) clones from *Jak2*WT and *Jak2*V617F genetic backgrounds were incubated for 24 h in low-supplemented RPMI-1640. RNA was isolated using a NucleoSpin RNA kit (Macherey-Nagel, Düren, Germany) following the manufacturer’s protocol. RNA quality was assessed via NanoDrop spectrophotometer (Thermo Fisher Scientific, Waltham, USA). RNA sequencing (RNAseq) was performed by NovoGene (NovoGene Germany, Munich, Germany) with an Illumina NovaSeq X Plus Sequencing System using a paired-end 150 bp read length sequencing strategy and a depth of at least 6G of data per sample. RNAseq analysis was performed using NovoMagic (NovoGene Germany, Munich, Germany) and R 4.5.1 (R Foundation for Statistical Computing, Vienna, Austria) [27]. Data was filtered to remove indeterminable, low-quality reads and adapters used in sequencing via fastp (1.0.1). HISAT2 (2.2.1) was used to align sequences with reference genome (mm39). Finally, featureCounts (2.0.6) was used to count the number of reads mapped to each gene. To investigate specific metabolic and stress–response adaptations, we used a targeted panel of genes comprising established markers for glycolysis, oxidative phosphorylation, amino acid metabolism, fatty acid metabolism, DNA damage response, apoptosis, and cell cycle. The selection of these markers was based on their established roles within their respective pathways. Heatmaps were generated using the pheatmap package in R 4.5.1 on log2-transformed FPKM values, scaled by row to highlight relative expression differences across genotypes [28]. To visualize the relationship between differentially expressed genes and significantly enriched biological processes, chord plots were generated using R 4.5.1 and the circlize package [29]. Input data consisted of differential expression values (log2 fold change) and GO enrichment results. To avoid overplotting, the visualization was restricted to the top 10 most significant GO terms (ranked by adjusted p-value). From the genes associated with these terms, the top 40 genes exhibiting the highest absolute log2 fold change were selected.

### Reverse transcriptase quantitative PCR (RT-qPCR)

RNA was isolated from 1 × 10^6^ cells using the NucleoSpin RNA kit (Macherey-Nagel, Düren, Germany) following manufacturer’s instructions. Reverse transcription was performed using 1 µg RNA using M-MLV Reverse Transcriptase (Thermo Fisher Scientific, Waltham, USA). RT-qPCR was performed with PowerTrack™ SYBR Green Master Mix on a 7500 Fast Real-time PCR System (Thermo Fisher Scientific, Waltham, USA). Primer sequences are listed in Table S3. Relative gene expression was calculated using the 2^−ΔΔCt^ method, normalized to *Hprt* as housekeeping gene, and expressed as log2 fold change relative to *Jak2*WT GLUT1/3 WT controls.

### BrdU-7AAD cell cycle staining

32D GLUT1/3 WT and double KO clone cells of both genetic backgrounds were seeded on a 6-well plate in 3 mL of low-supplemented RPMI for 24 h (3 × 10^5^ initial cells). After 22 h, 20 µM 5’-bromo-2’-deoxyuridine (BrdU, Thermo Fisher Scientific, Waltham, USA) were spiked in. Cells were cultured for two more hours at 37 °C, 5% CO_2_. After harvesting, cells were transferred into FACS tubes and washed once with a wash buffer consisting of 1x PBS supplemented with 2% FCS. Cells were then fixed by resuspending pellets in 100 µL of Reagent A from FIX & PERM™ kit (Thermo Fisher Scientific, Waltham, USA) and incubated for 3 min at RT. Next, 3 mL ice-cold methanol were added, and mixed by vortexing. After another 10 min incubation on ice, cells were washed twice with wash buffer. Pellets were resuspended in 100 µL of DNase I solution, diluted in 1x PBS (~ 40 Units/mL, Thermo Fisher Scientific, Waltham, USA). Cells were then incubated for 1 h at 37 °C. After another wash step, pellets were resuspended in 100 µL of Reagent B from the FIX & PERM™ kit. 2 µL of Anti-BrdU APC antibody, sourced from an eBioscience™ BrdU Staining Kit for Flow Cytometry (Thermo Fisher Scientific, Waltham, USA), were added to each tube and cells incubated for 30 min at RT in dark. After a final washing step, pellets were resuspended in 400 µL Wash buffer and stained with 1 µL 7-AAD (0.05 mg/mL) for 15 more minutes at RT in dark. Samples were then immediately analyzed on a Gallios flow cytometer (Beckmann Coulter, Brea, USA), without an additional washing step.

### Murine *Jak2*V617F knock-in model treatment with pharmacological inhibitors

CD45.1^+^ B6.SJL-Ptprca Pepcb/BoyJ (Pep Boy) mice were purchased from Charles River and used as recipients in this study (Charles River, Wilmington, USA). A *Jak2*V617F KI C57BL/6 *Jak2*^+/fl^ *x Vav-iCre* mouse model previously established by Villeval and Plo was bred and used as BM donor [30, 31]. Recipient Pep Boy CD45.1^+^ mice were lethally irradiated with 2 × 4 Gy, staggered by 4 h, and transplanted the same day with BM cells from either *Jak2*^+/fl^ *Vav-iCre*^+^ or *Jak2*^+/fl^ *Vav-iCre*^−^ donors, establishing *Jak2*V617F and *Jak2*WT groups, respectively.

All treatments were administered via intraperitoneal (i.p.) injection and with a volume of 10 µL/g body weight. *Jak2*WT mice and a control *Jak2*V617F mice group received daily vehicle (0.2% DMSO/PBS). Three *Jak2*V617F groups were treated with one of the following inhibitors: echinomycin (Ech, Merck, Darmstadt, Germany), an inhibitor of HIF-1 that intercalates DNA at hypoxia-response elements upstream of HIF–regulated genes, dissolved in DMSO/DPBS and administered daily at a dosage of 10 µg/kg body weight; acriflavine (ACF, Sigma-Aldrich, St. Louis, USA), inhibiting HIF-1α dimerization with HIF-1β, dissolved in PBS and administered daily at 8 mg/kg bodyweight; and KL-11743 (Sigma-Aldrich, St. Louis, USA), a GLUT1/2/3/4 inhibitor. Initially dissolved in H_2_O with 0.5% Methylcellulose (Sigma-Aldrich, St. Louis, USA) and 0.25% Tween-80 (Sigma-Aldrich, St. Louis, USA), the formulation was adjusted in week 6 to 10% DMSO (Serva, Heidelberg, Germany), 40% PEG-300 (MedChemExpress, Monmouth Junction, USA) and 50% H_2_O due to solubility issues. KL-11743 was freshly prepared and administered at 100 mg/kg body weight every 48 h.

Blood was drawn via tail vein at weeks 2 and 4 post-transplantation to monitor engraftment and disease progression by flow cytometry for CD45.1^+^ and CD45.2^+^ cells. After treatment initiation, blood draws were performed weekly until study endpoint. Hematologic parameters were assessed using an Element HT5™ Hematology Analyzer (Antech Diagnostics, Viernheim, Germany). At study endpoint, BM, spleen, and peripheral blood (PB) were collected and analyzed by flow cytometry using the antibodies listed in Table S4 on either a Gallios Flow Cytometer (Beckmann Coulter, Brea, USA) or BD FACSCanto™ II (BD, East Rutherford, USA). Data was analyzed using FlowJo v10.10.0 (BD, East Rutherford, USA).

### Primary patient material

Blood samples from MPN patients or healthy donors (HD) were obtained from the Department of Hematology, Oncology, Hemostaseology, and Stem Cell Transplantation and the Institute for Transfusion Medicine und Cell Therapeutics at the University Hospital Aachen. Samples were diluted 1:3 with PBS and carefully layered onto 15 mL Pancoll human (PAN-Biotech, Aidenbach, Germany) and centrifuged at 400 xg for 30 min at room temperature, with gentle acceleration and deceleration. Peripheral blood mononuclear cells (PBMCs) were collected from the interphase, washed twice with PBS, and resuspended for downstream assays.

For proliferation assays, 1 × 10^5^ PBMCs were seeded in 96-well flat-bottom plates in IMDM (Thermo Fisher Scientific, Waltham, USA) supplemented with 10% FCS, 1% penicillin/streptomycin with hSCF (50 ng/mL), hFLT3L (50 ng/mL), hIL-3 (10 ng/mL) and hIL-6 (10 ng/mL, ImmunoTools, Friesoythe, Germany). Cells were cultured for 7 days with different inhibitors or DMSO (0.02%) as vehicle control. Culture media was not replenished over the course of the assay, and no additional dosage of inhibitors were added beyond day 0. The inhibitors included acriflavine (Sigma-Aldrich, St. Louis, USA), a HIF-1α inhibitor; Glutor (Sigma-Aldrich, St. Louis, USA), a GLUT1/2/3 inhibitor; and KL-11743 (Sigma-Aldrich, St. Louis, USA), a GLUT1/2/3/4 inhibitor, each at 1 µM [32–34]. Glutor and KL-11743 were also combined with CB-839 (0.5 µM, Sigma-Aldrich, St. Louis, USA) to assess potential redundancy in glutaminase pathways [35]. After 7 days of incubation at 37 °C and 5% CO_2_, living and dead cells were counted using a Neubauer chamber with trypan blue (Carl ROTH, Mannheim, Germany).

For colony-forming unit (CFU) assays, PBMCs were seeded in human methylcellulose (Methocult H4230, Stemcell Technologies, Vancouver, Canada) supplemented with 20% IMDM, 1% penicillin/streptomycin (Thermo Fisher Scientific, Waltham, USA), hSCF (50 ng/mL), hIL-3 (10 ng/mL), hGM-CSF (10 ng/mL), and hEPO (14 ng/mL, ImmunoTools, Friesoythe, Germany). 1 × 10^6^ cells per condition were mixed with either DMSO (0.02%), Glutor (1 µM, Sigma-Aldrich, St. Louis, USA) or KL-11743 (1 µM, Sigma-Aldrich, St. Louis, USA), and seeded in 35 mm gridded dishes (Corning, Corning, USA). After 10–14 days of incubation at 37 °C and 5% CO_2_, colonies were counted by brightfield microscopy.

### Statistical analysis

Pairwise comparisons were performed using Mann-Whitney tests. Multiple comparisons were performed via one-way or two-way ANOVA, as appropriate, followed by Dunnett’s, Bonferroni’s, Tukey’s or Šídák post-hoc tests, using GraphPad Prism 10.6.1 (GraphPad Software, Boston, USA).

## Results

### *Jak2V617F* increases metabolic activity in a HIF-1α–dependent manner

**Fig. 1:**
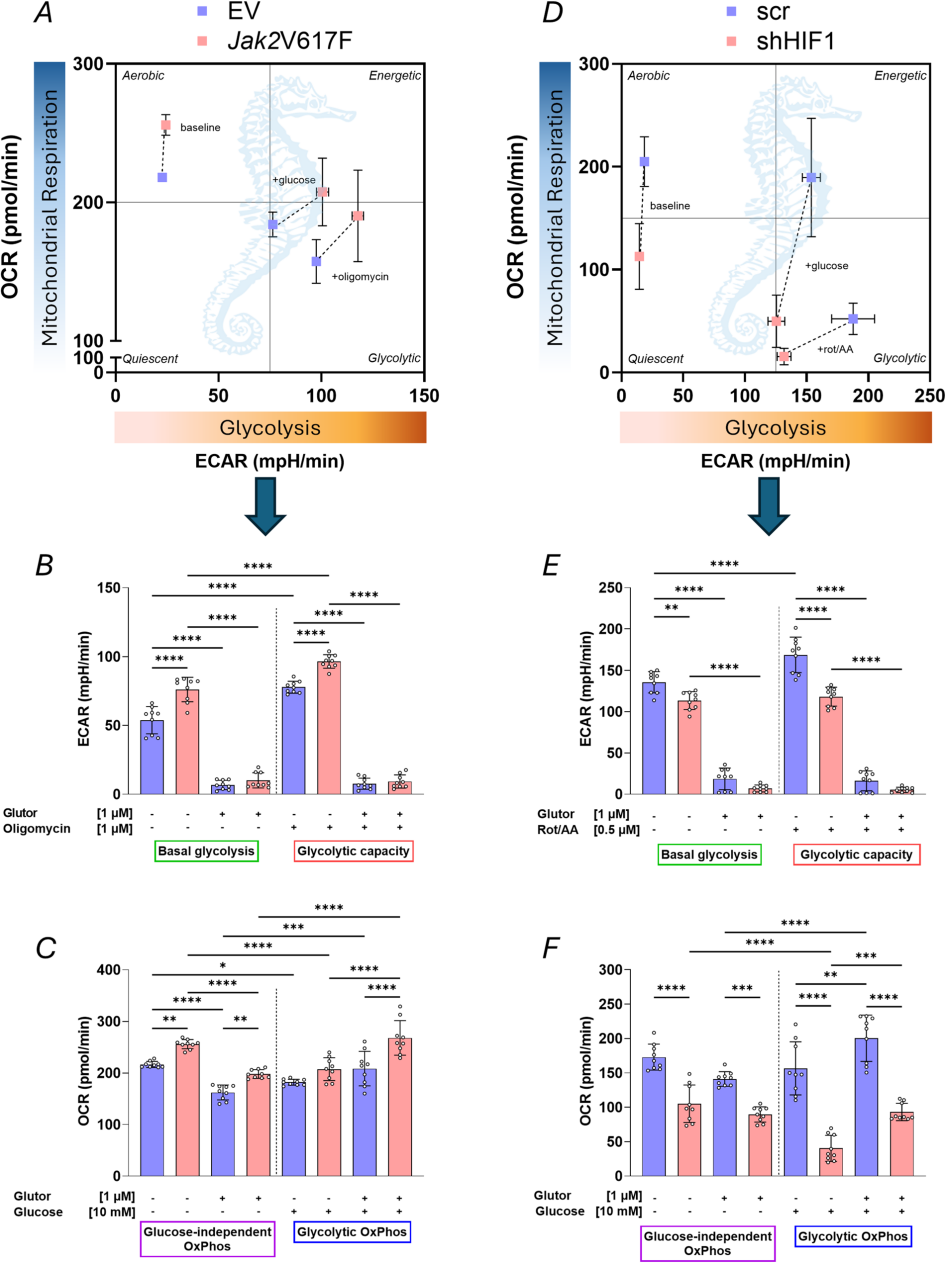
*Jak2V617F *increases metabolic activity and capacity in 32D cells in a HIF-1–dependent manner.** A** 2D plot of metabolic activity of EV and *Jak2*V617F 32D cells based on extracellular flux measured over the assay course. Quadrants were added to visualize differences in metabolic states (*n* = 3). **B** Changes in extracellular acidification rate (ECAR), corresponding to (**A**), from basal glycolysis (10 mM glucose, left) to maximal glycolytic capacity (right) after addition of oligomycin (1 µM) as mitochondrial complex V inhibitor. **C** Changes in oxygen consumption rate (OCR), corresponding to (**A**), in absence of glucose (left) compared to basal glycolysis (right) after addition of glucose (10 mM). **D** 2D plot of 32D *Jak2*V617F–expressing short hairpin RNA targeting *Hif1α* or scrambled controls (scr) (**A**). Rotenone/antimycin A were used instead of oligomycin (*n* = 3). **E** ECAR changes of 32D *Jak2*V617F scr and shHIF-1 α cells, corresponding to (**D**). Rotenone/antimycin A (0.5 µM each) were used to inhibit mitochondrial complex I and III. **F** OCR changes of 32D *Jak2*V617F scr and sh*Hif1α* cells, assayed in separate phases, corresponding to (**D**). Rotenone/antimycin A (0.5 µM each) were used to inhibit mitochondrial complex I and III. Cells were incubated with either DMSO (0.02%) or Glutor (1 µM) for 1 h before assay. All data are presented as mean ± SD. Statistical analysis was performed using ordinary one-way ANOVA with Šídák post-hoc test, comparing conditions differing by no more than one variable (genetic background, pretreatment, metabolic state). Significant p-values are indicated as follows: *< 0.05, **< 0.01; ***< 0.001, ****< 0.0001 (*n* = 3, 3 measured timepoints per phase per n, each timepoint averaged from 8 analytical replicates per condition)

We previously reported that HIF-1–regulated key glycolytic enzymes are upregulated in *Jak2*V617F–expressing 32D cells and that their expression can be reduced by either pharmacologic inhibition or knockdown of HIF-1 [10]. Based on these findings, we analyzed cellular metabolism in a *MPL*WT *Jak2*V617F 32D cell line using extracellular flux (Seahorse) assays. Compared with empty vector (EV) controls, *Jak2*V617F–expressing cells exhibited significantly elevated glycolytic flux and mitochondrial respiration, as reflected by increased extracellular acidification rate (ECAR) and oxygen consumption rate (OCR) (Fig. 1A, B, C).

Increased ECAR indicates enhanced lactate production and export, while elevated OCR reflects increased oxidative phosphorylation (OXPHOS) activity. Basal glycolysis and maximal glycolytic capacity were both increased in *Jak2*V617F cells compared with EV controls (Fig. 1B). OCR was likewise elevated in *Jak2*V617F–positive cells, before glucose addition, but reduced in response to glucose addition (Fig. 1C). This rapid, glucose-induced suppression of OXPHOS is characteristic of the Crabtree effect and demonstrates a pronounced shift toward aerobic glycolysis in the mutated cells. The high basal glycolytic activity was mirrored by increased OXPHOS, with a decrease in OCR upon glucose addition to the assay medium, consistent with glucose–driven metabolic reprogramming. However, OCR remained significantly higher in *Jak2*V617F cells under all conditions.

Pharmacological inhibition of glucose transport using 1 µM of the GLUT1/2/3–targeting piperazine-2-one derivative Glutor resulted in near–complete attenuation of glycolytic flux, as demonstrated by a pronounced reduction in ECAR (Fig. 1B), confirming a strong dependence on glucose uptake for maintaining the elevated metabolic state. In turn, Glutor increased OCR in *Jak2*V617F cells, likely due to compensatory OXPHOS utilizing other metabolic pathways to generate substrate, such as β-oxidation and glutamine (Fig. 1C), whereas effects of Glutor on *Jak2*WT were limited. Full time-course curves depicting the complete kinetics of ECAR and OCR measurements throughout the assay are illustrated in Figure S1A.

For subsequent Seahorse experiments, oligomycin was replaced by a combination of rotenone and antimycin A (0.5 µM each), as oligomycin failed to reliably suppress OCR in 32D cells. Using previously generated *Jak2*V617F sh*Hif1α* 32D cells, we investigated the effect of reduced HIF-1α levels on cellular metabolism via *Hif1α* KD (Fig. 1D-F) [10]. Compared with scrambled controls (scr), *Hif1α* KD cells resulted in a marked reduction of both ECAR and OCR approaching levels observed in *Jak2*WT cells. This metabolic reversion mirrored the differences observed between EV and *Jak2*V617F cells in Fig. 1A-C. Addition of Glutor to *Hif1α* KD cells produced effects comparable to those observed in *Hif1α*–competent cells, arguing against compensatory upregulation of alternative glucose transporters in response to reduced HIF-1 signaling in *Jak2*V617F–positive 32D cells. Full ECAR and OCR kinetics are shown in Figure S1B.

Collectively, these results demonstrate that *Jak2*V617F drives a glucose–centered metabolic phenotype, marked by elevated glycolysis and mitochondrial respiration, which is strongly dependent on HIF-1. KD of *Hif1a* effectively reverts *Jak2*V617F–positive cells toward a more quiescent, less glycolytic metabolic state.

### Knockout of *Slc2a1* and *Slc2a3* reveals functional redundancy and glucose addiction in *Jak2*V617F–mutated 32D cells

**Fig. 2:**
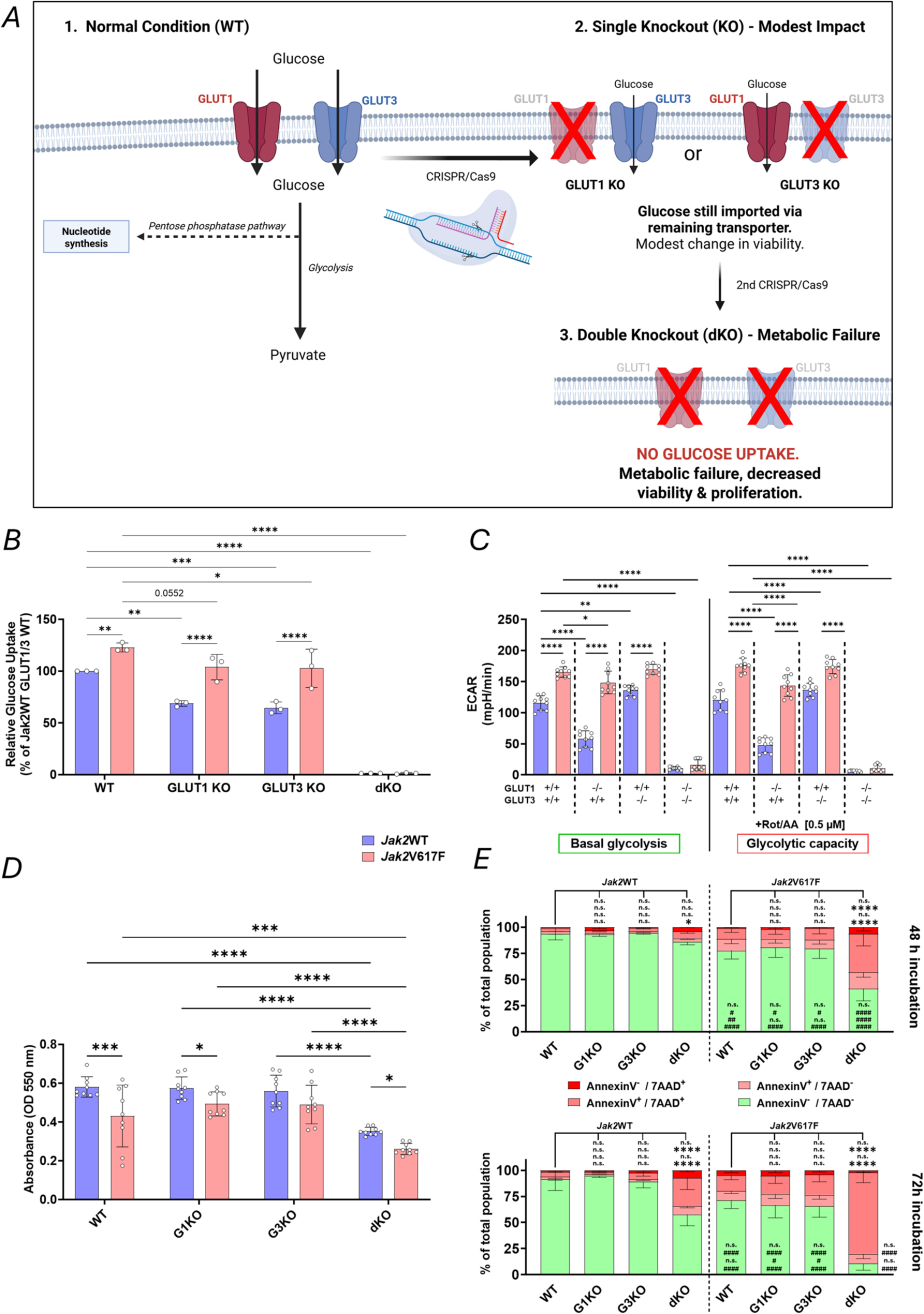
*Jak2V617F *induces functional redundancy between GLUT1 and GLUT3, revealing a metabolic vulnerability upon dual loss.** A** Schematic illustration of glucose transport via GLUT1 and GLUT3 and the CRISPR–Cas9–mediated knockout strategy. In the absence of GLUT-mediated glucose uptake, downstream glucose–dependent metabolic pathways are deprived of substrate. **B** Fold change in glucose uptake, measured by luminescence, across all 32D GLUT KO clones generated in *Jak2*WT (blue) and *Jak2*V617F (red) backgrounds. Values are normalized to the *Jak2*WT GLUT WT group (*n* = 3, three independent clones of each condition served as analytical triplicates for each independent experiments). **C** ECAR flux changes of all 32D GLUT WT and KO clones in both genetic backgrounds during basal glycolysis (left) and the maximum glycolysis capacity (right) following inhibition of mitochondrial complex I and III via rotenone (Rot)/antimycin A (AA; 0.5 µM each). Statistical analysis was performed using ordinary one-way ANOVA with Šídák multiple comparison tests within and between metabolic phases. Each clone per condition was measured once as independent experiment (*n* = 3, 3 measured timepoints per phase per n, each timepoint averaged from 8 analytical replicates per condition). **D** Absolute absorbance values from MTT assays for all GLUT WT and KO clones from both genetic backgrounds. Data are shown as raw absorbance values. (*n* = 3, analytical triplicates per clone per n). **E** Proportions of cells in distinct apoptosis stages displayed as stacked columns. Living (Annexin V (AnnV)-, 7AAD-), early apoptotic (AnnV+, 7AAD-), late apoptotic (AnnV+, 7AAD+) and necrotic (AnnV-, 7AAD+) cells after 48 h (top) and 72 h (bottom) with physiologically supplemented culture medium. P-values at the bottom of bar graphs (#) correspond to comparisons between *Jak2* genetic backgrounds (*n* = 3, each clone measured per n and results averaged). Unless otherwise stated, statistical analyses were performed using ordinary two-way ANOVA with Tukey’s post-hoc test. All data are presented as mean ± SD. Statistical significance is indicated as follows: *< 0.05, **< 0.01; ***< 0.001, ****< 0.0001

As glycolysis fuels multiple anabolic pathways favored by oncogenic proliferation, we sought to genetically ablate glucose uptake in our 32D *MPL*WT *Jak2*V617F cell line model, using 32D *MPL*WT *Jak2*WT cells as control (Fig. 2A).

Based on our earlier observation that the GLUT1/2/3 inhibitor Glutor almost completely suppressed glucose uptake (Fig. 1A, B, D, E), we targeted members of the Slc2a family inhibited by this compound. As *Slc2a2* expression is largely restricted to hepatocytes, we focused on *Slc2a1* (GLUT1) and *Slc2a3* (GLUT3). Using CRISPR-Cas9, we generated single (*Slc2a1* or *Slc2a3*) and double KO (dKO) clones in both *Jak2*V617F and *Jak2*WT genetic backgrounds (Fig. 2A). For each genotype and KO condition, three independent clones were established to ensure reproducibility. Successful gene disruption was confirmed by sequencing and immunoblotting (Fig. S2A). Due to poor performance of the GLUT3 antibody, resulting in unspecific binding, KO of GLUT3 was determined via Sanger sequencing and validated by functional assays (Fig. 2B and C). Detailed information on the sequence alterations in all generated clones is provided in Table S5.

Consistent with our metabolic profiling, *Jak2*V617F cells exhibited increased glucose uptake compared to *Jak2*WT cells (Fig. 2B), in line with previous reports [36]. Single KO of either GLUT1 or GLUT3 resulted in only modest reduction in glucose uptake in both genetic backgrounds, indicating functional redundancy between these transporters. In contrast, glucose uptake was completely abrogated in the dKO clones of both *Jak2*WT and *Jak2*V617F cells, supporting the conclusion that no additional glucose transporters are expressed at functionally relevant levels in 32D cells or induced by JAK2 oncogenic signaling.

Extracellular flux analyses further corroborated transporter redundancy between GLUT1 and GLUT3 in the *Jak2*V617F background (Fig. 2C). Loss of GLUT1 caused a significant, but mild reduction in basal ECAR and maximal glycolytic capacity in *Jak2*V617F cells. In contrast, *Jak2*WT GLUT1 KO cells displayed a pronounced reduction in ECAR, an effect not proportionally reflected in the glucose uptake assay (Fig. 2B). This discrepancy could indicate compensation via GLUT3–mediated glucose uptake in *Jak2*WT cells, or, alternatively, reduced glycolytic flux downstream of glucose import, potentially due to altered expression or activity of glycolytic enzymes (Fig. 2C).

Interestingly, *Jak2*WT GLUT3-KO cells exhibited increased ECAR compared to GLUT WT cells, suggesting compensatory metabolic adaptations. As expected, both *Jak2*WT and *Jak2*V617F dKO cells displayed near-complete loss of ECAR, further supporting the absence of alternative glucose uptake mechanisms.

OCR measurements confirmed elevated metabolic activity in *Jak2*V617F GLUT WT and single KO clones compared with *Jak2*WT clones (Fig. S3A). In contrast, *Jak2*WT GLUT1 KO displayed a reduced metabolic profile, consistent with the ECAR measurements. GLUT1/3 dKO cells displayed overall suppressed mitochondrial activity, as indicated by reduced OCR, independent of glucose availability. Notably, glucose addition decreased OCR across all conditions, suggesting that glycolytic metabolites are preferentially shunted into anabolic pathways rather than oxidized in the mitochondria. Complete OCR and ECAR time-course measurements, including baseline respiration rates, are provided in Fig. S3B.

To assess the functional consequences of glucose transporter loss under physiologically relevant conditions, cells were cultured in RPMI-1640 supplemented with 10% serum, murine IL-3 (2 ng/mL), low glucose (5 mM), and low glutamine (1 mM). These concentrations were chosen to approximate physiological ranges rather than the supraphysiological nutrient concentrations typically used in standard culture conditions. Sodium-pyruvate was deliberately omitted, as pyruvate uptake does not require GLUT1 or GLUT3 and could therefore mask glucose transporter–dependent phenotypes.

Metabolic activity was quantified via MTT assays, revealing a significant reduction in *Jak2*V617F cells under these nutrient–limited conditions, likely reflecting reduced proliferation, even in GLUT-WT clones (Fig. 2D). The differences between *Jak2*WT and *Jak2*V617F backgrounds were most pronounced in GLUT WT, GLUT1 KO, and dKO cells. In both *Jak2* backgrounds, combined loss of GLUT1 and GLUT3 significantly reduced metabolic activity, consistent with impaired proliferative capacity. The heterogeneity observed among *Jak2*V617F GLUT WT clone growth rate was not recapitulated in independent proliferation assays, which showed a more homogenous growth kinetics. These experiments confirmed the proliferative advantage of *Jak2*WT GLUT WT cells and highlighted the pronounced impact of GLUT1/3 dKO on proliferation and viability (Fig. S2B).

To determine whether the reduced proliferation and viability was associated with increased apoptosis, cells were stained with Annexin V and 7-AAD and quantified after 48 and 72 h of culture under approximately physiological conditions (Fig. 2E). At 48 h, *Jak2*V617F cells exhibited a baseline level of apoptotic markers not observed in *Jak2*WT cells suggesting that *Jak2*V617F cells are more dependent on glucose. This *Jak2* background–dependent difference was most pronounced in the *Jak2*V617F GLUT1/3 dKO cells, with more than 50% being positive for Annexin V, 7-AAD, or both, whereas *Jak2*WT GLUT1/3 dKO cells showed only a modest increase in apoptosis. After 72 h, apoptotic rates increased in *Jak2*WT dKO cells and all *Jak2*V617F genotypes. However, *Jak2*V617F dKO cells were most severely affected, with fewer than 10% of cells remaining viable, underscoring their extreme sensitivity to loss of glucose uptake.

To corroborate the effects of GLUT inhibition in other models, we employed SET-2 and HEL cells, both human JAK2V617F-positive post-MPN AML cell lines, and investigated their response to varying concentrations of Glutor, either as a single agent or in combination with 500 nM ruxolitinib. We observed that targeted single-agent GLUT inhibition successfully reduced the global metabolic activity of SET-2 cells within the nanomolar range (Fig. S2C). This sensitivity was particularly pronounced under nutrient-restricted conditions (low-supplemented medium), highlighting a severe vulnerability to glucose deprivation when exogenous nutrients are scarce. HEL and 32D cells also responded to single-agent Glutor treatment, although higher concentrations were required to achieve a comparable reduction in metabolic viability.

Interestingly, the response to co-treatment diverged significantly depending on the cellular model. In 32D *Jak2*V617F cells, combining Glutor with ruxolitinib produced the anticipated synergistic therapeutic benefits, resulting in a profound reduction in metabolic viability. In stark contrast, across the human post-MPN AML models (SET-2 and HEL), the combination proved antagonistic. These cells displayed either a similar or higher relative metabolic viability compared to the expected cumulative effect of the single agents. This suggests that, specifically in these advanced human models, the ruxolitinib–induced reduction in baseline metabolic demand inadvertently protects the cells from targeted energy starvation.

### Transcriptomic analysis reveals *Jak2*V617F-specific vulnerability to glucose deprivation

**Fig. 3:**
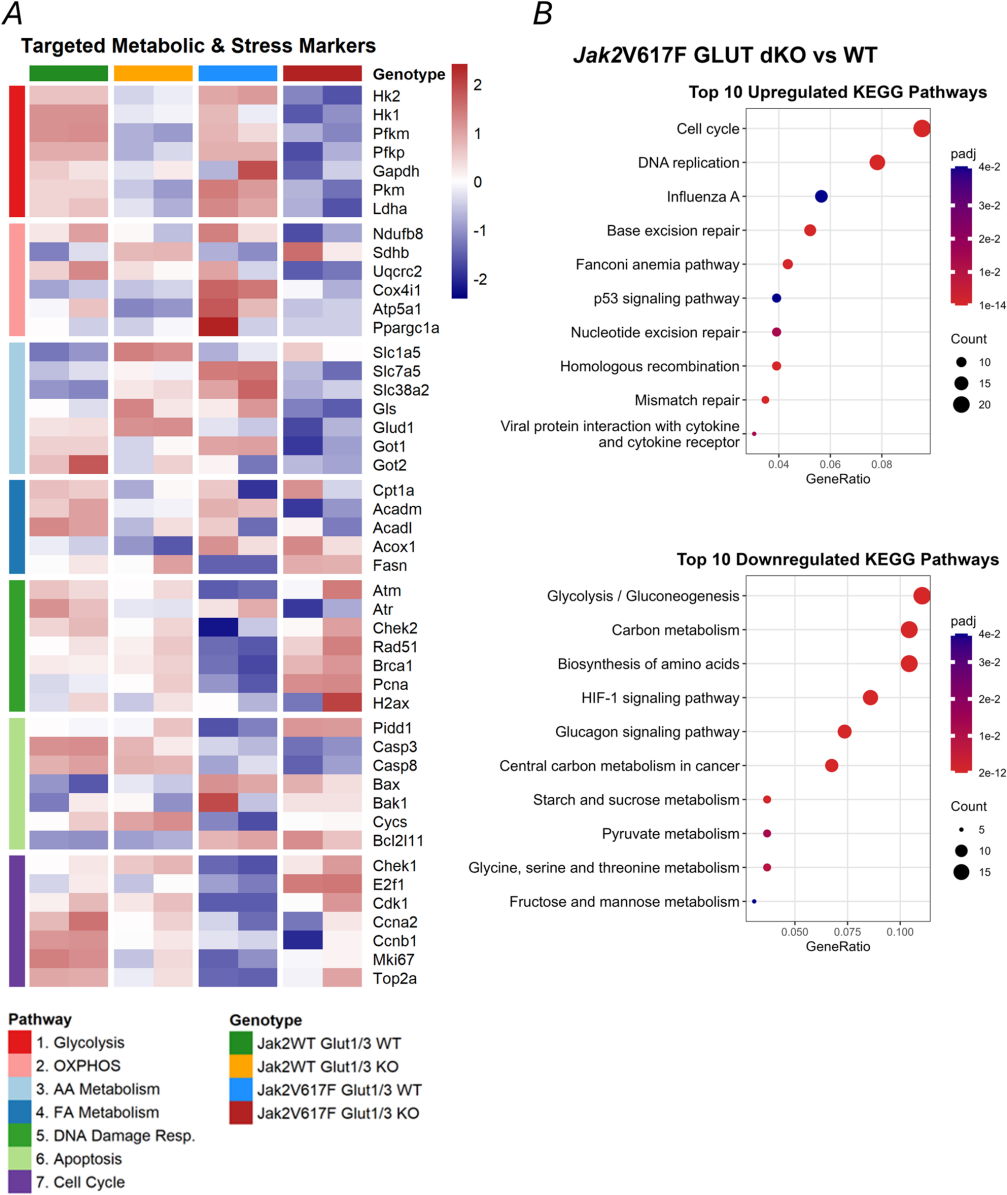
RNA-Seq of 32D GLUT1/3 dKO clones reveal inability to overcome loss of GLUT1/3 in *Jak2*V617F-positive cells.** A** Heatmap showing normalized expression of key genes from curated metabolic and stress marker gene sets across indicated groups. Rows correspond to individual genes, clustered according to their gene sets. Gene expression values are scaled per gene, with higher expression depicted in red and lower in blue. **B** KEGG enrichment analysis of differential gene analysis comparing *Jak2*V617F GLUT dKO vs. WT RNA sequencing, shown as dot plots of top 10 KEGG pathways up (top) and downregulated (bottom) pathways in *Jak2*V617F GLUT dKO cells. Thresholds for analysis were set at an adjusted p-value = 0.05 and Benjamin-Hochberg method used for multiple testing corrections

To define the transcriptional consequences of impaired glucose uptake, bulk RNA-seq was performed on GLUT1/3 dKO and WT clones from *Jak2*WT and *Jak2*V617F genetic backgrounds. Overall gene expression distributions were comparable across samples, indicating consistent library quality and sequencing depth (Fig. S4A). Principal component analysis demonstrated clear separation of samples by genotype (Fig. S4B), which was further supported by Pearson correlations (Fig. S4C). Differential gene expression between *Jak2*V617F GLUT1/3 dKO and WT cells is summarized in a volcano plot (Fig. S5A). In addition to the expected strong attenuation of HIF-1 signaling, glycolysis, carbon metabolism, and amino acid biosynthesis, RNAseq revealed pronounced dysregulation of cell cycle regulation, DNA replication, and DNA repair pathways in *Jak2*V617F GLUT1/3 dKO cells compared with *Jak2*V617F GLUT WT controls (Fig. 3A + B, Fig. S5B).

**Fig. 4:**
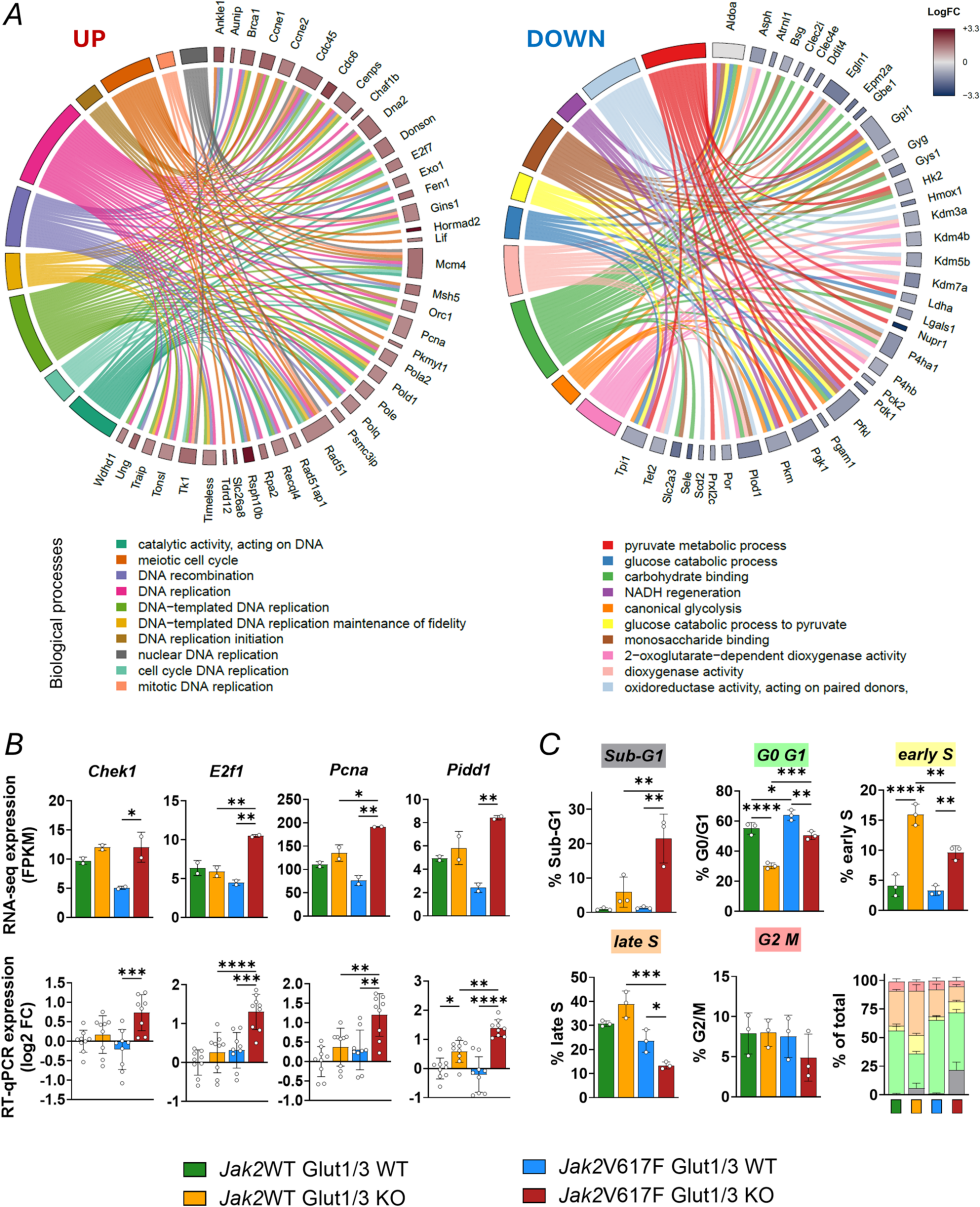
GLUT1/3 ablation induces replication stress and S-phase–mediated apoptosis in Jak2V617F cells.** A** The plot shows the linkage between the top 10 enriched biological processes (left) and the top 40 significantly regulated genes (right) in *Jak2*V617F GLUT dKO vs. WT samples. Connecting ribbons are colored by GO term. The outer ring displays the Log2 Fold Change of each gene (Blue: Downregulated; Red: Upregulated; Grey: Neutral). Visualizations were generated using the circlize package in R. **B** Validation of expression of selected genes from RNA-sequencing (top) through RT-qPCR (bottom). Sequencing data was normalized to fragments per kilobase million (FPKM) (*n* = 2). RT-qPCR data was normalized to *Hprt* expression, relative to *Jak2*WT GLUT1/3 WT (*n* = 3, three independent clones of each condition served as triplicates for each independent experiment). **C** Cell cycle analysis in 32D *Jak2*WT vs. *Jak2*V617F GLUT WT and dKO clones, assessed via BrdU/7-AAD staining (*n* = 3, three independent clones of each condition per n). Statistical analysis was performed using ordinary one-way ANOVA with Šídák multiple comparison tests, comparing all groups not differing by more than one genetic variable. Significant p-values are indicated as follows: *< 0.05, **< 0.01; ***< 0.001, ****< 0.0001. RNA-seq data originates from 2 clones per group

Given these findings and the metabolic constraint imposed by GLUT1/3 deletion, we hypothesized that *Jak2*V617F cells might engage compensatory survival mechanisms or stress-induced arrest. Surprisingly, targeted expression analysis of canonical pathway markers revealed no compensatory upregulation of the alternative metabolic pathways, including OXPHOS, amino acid (AA), and fatty acid (FA) metabolism (Fig. 3A). Instead, dKO cells exhibited profound downregulation of OXPHOS and AA metabolism genes, indicative of metabolic collapse rather than adaptation. This failure to switch fuel sources to lipids and amino acids, despite their supplementation in the media, coincided with a cellular crisis: *Jak2*V617F GLUT1/3 dKO clones showed elevated expression of apoptosis– and replication stress–associated genes corroborating our findings from the apoptosis and viability assays (Fig. 2D and E). While mitogenic gene expression indicated a strong drive for proliferation, this signature more likely reflects an accumulation of cells stalled at the G1/S metabolic checkpoint, as the constraints imposed by GLUT1/3 depletion prevented successful cell division (Figs. 2E and 4A).

To investigate this paradox, we analyzed signatures of replication stress and cell cycle arrest (Fig. S6). We observed a marked upregulation of these genes in *Jak2*V617F GLUT1/3 dKO cells, whereas expression remained unchanged in *Jak2*WT cells. This suggests that constitutive JAK2V617F signaling actively drives metabolically starved cells into a stressed, arrested state, rather than allowing them to enter quiescence.

Interestingly, the *Jak2*V617F GLUT1/3 WT clones displayed a distinct transcriptional phenotype compared with *Jak2*WT cells, with a global downregulation of stress pathways, while OXPHOS and amino acid metabolism-associated genes were elevated (Figs. 3A and B and 4A). We propose that, in line with our MTT and proliferation assay findings, heightened glucose dependency driven by *Jak2*V617F induces a protective state under glucose-limited culture conditions, suppressing proliferation and metabolic demand to maximize nutrient uptake and preserve viability. In contrast, complete loss of glucose transport in dKO cells precipitated terminal stress and cell death.

To validate RNAseq findings, stress–associated genes were prioritized by ranking transcripts first by adjusted p-value and subsequently by fold change, focusing on genes with established roles in stress-response pathways. Independent RT-qPCR validation confirmed that GLUT1/3 dKO induced robust upregulation of *Chek1*, *E2f1*, *Pcna*, and *Pidd1* in *Jak2*V617F clones, consistent with the RNAseq results (Fig. 4B). To functionally validate the cell cycle alterations predicted by our RNA-seq and RT-qPCR data, we performed BrdU/7-AAD cell cycle analyses on our 32D *Jak2*WT and *Jak2*V617F GLUT1/3 WT and dKO clones (Fig. 4C; gating strategy in Fig. S7). We found that deletion of GLUT1/3 led to a significant accumulation of cells in the early S-phase across both Jak2 backgrounds. However, while this accumulation persisted into the late S-phase in *Jak2*WT cells, *Jak2*V617F dKO cells exhibited a marked depletion of the late S-phase population alongside a concurrent increase in the sub-G1 fraction. This prominent sub-G1 population indicates a transition to apoptosis during S-phase, likely driven by metabolic stress-induced replication failure, and perfectly corroborates our apoptosis assay data.

Together, the findings obtained from the GLUT1/3 KO analyses demonstrate that *Jak2*V617F–positive cells compensate for the loss of either GLUT1 or GLUT3 through functional redundancy but exhibit profound sensitivity to complete abrogation of glucose import. While disruption of glucose uptake reduces proliferation in both *Jak2*WT and *Jak2*V617F cells, the resulting metabolic stress preferentially induces apoptosis in *Jak2*V617F–mutated cells, providing strong evidence for glucose addiction driven by oncogenic JAK2 signaling.

### *Jak2*V617F–driven MPN exhibits resistance to metabolic targeting in vivo despite robust in vitro sensitivity

Based on our previous findings and the in vitro data obtained from glucose transporter KO clones, pharmacological inhibition of HIF-1α and glucose transporters emerged as a potential strategy to selectively target *JAK2*V617F–positive cells in MPN. To assess the translational relevance of this approach, we conducted an in-vivo intervention study employing a *Jak2*V617F-floxed *Vav*-i*Cre* mice model previously established by Villeval and Plo [30, 31]. *Jak2*^VF/+^ x *Vav-iCre*^+^ mice develop a PV–like phenotype due to Cre–mediated recombination of *Jak2* Exon 13 [37]. An overview of the experimental design is shown in Fig. S8A. Four weeks post transplantation, mice were randomly assigned to the treatment groups (vehicle, Ech, ACF, and KL-11743) and treatments were initiated. Mice treated with Ech, an inhibitor of HIF-1 signaling that intercalates with DNA at hypoxia-response elements (HRE) upstream of HIF-regulated genes, showed a heterogenous response across erythroblast populations in PB, BM, and spleen (Fig. S8E). While some animals showed marked increases, others exhibited reductions toward *Jak2*WT levels that were partially reflected in corresponding hemogram parameters. In contrast, treatment with acriflavine (ACF), a HIF-1α inhibitor that binds to the HIF-1α subunit in the cytoplasm and prevents dimerization with HIF-1β in the nucleus resulted in a trend toward reduced proerythroblast (*p* = 0.0593) and basophilic/polychromatic/ortho-chromatic–erythroblast (*p* = 0.0813) populations in the BM, accompanied by increased erythroblasts frequencies in the spleen (Fig. S8E). This pattern is compatible with partial suppression of erythropoiesis within the BM niche, potentially compensated by extramedullary erythropoiesis, thereby limiting measurable effects on PB parameters.

Similarly, mice treated with KL-11743, a GLUT1/2/3/4 inhibitor chosen due to low penetration of blood-brain barrier and proven effect against glucose–addicted solid tumors in in-vivo studies, exhibited erythroblast population patterns comparable to those observed in the ACF–treated group [33]. Combined basophilic, polychromatic and orthochromatic erythroblasts population was significantly reduced (*p* = 0.0140) in the BM, while proerythroblast populations showed a trend (*p* = 0.0721) toward increased representation in the spleen. Consistent with a treatment–induced shift of erythropoiesis from BM to spleen, HIF-1 inhibition was accompanied by a trend to increased circulating pro-erythroblasts, indicative of stress-associated and less compartmentalized extramedullary erythropoiesis [38].

Taken together, we observed only subtle and tissue–specific alterations in erythroid precursor populations, pharmacological inhibition of HIF-1 or glucose transporters failed to robustly ameliorate splenomegaly or normalize hematologic parameters in vivo. Under the experimental conditions tested, pharmacological inhibition of HIF-1 or glucose transporters did not result in measurable improvement of disease parameters in this murine model. These findings may reflect limitations in drug stability, delivery, bioavailability, target engagement, or compensatory hematopoietic adaptation. Therefore, while no therapeutic benefit was observed in this setting, further optimization of dosing, delivery, and combination strategies will be required before excluding their potential clinical relevance.

### In vitro targeting of HIF-1 and GLUT1/3 reduced proliferation and survival of patient–derived PBMCs

**Fig. 5:**
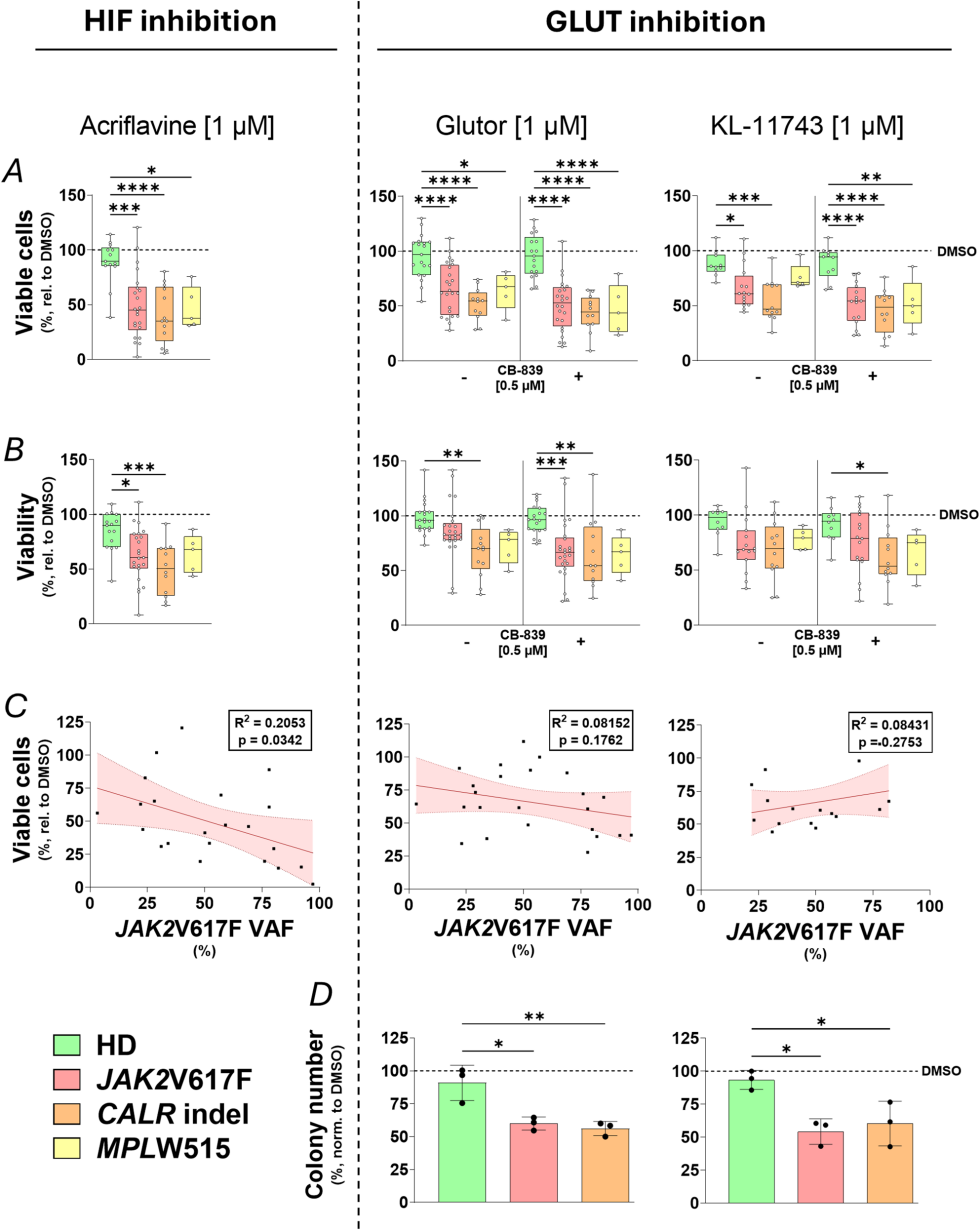
Patient–derived PBMCs are sensitive to HIF-1 and GLUT1/3 inhibition in vitro.** A** Proliferation of PBMCs isolated from healthy donors (HD) and MPN patients, normalized to the internal DMSO control of each sample. Following Ficoll separation, PBMCs were treated for 7 days with either acriflavine (ACF, 1 µM; left), Glutor (1 µM; middle), or KL-11743 (1 µM; right), either alone or in combination with the glutaminase inhibitor CB-839 (0.5 µM). Cell numbers were determined using a Neubauer chamber with Trypan blue exclusion to discriminate viable from dead cells. **B** Cell viability corresponding to samples in (**A**), calculated as the ratio of living to total cells and normalized to the DMSO control of each sample. Samples in (**A**) and (**B**) were stratified by the underlying driver mutations. Statistical comparisons were performed between patient– and HD–derived samples for each treatment condition. Combination treatments including CB-839 were additionally compared with their respective single-agent conditions. Statistical analysis was performed using ordinary one-way ANOVA with Dunnett’s (ACF) or Šídáks (Glutor, KL-11743) post-hoc correction. **C** Linear regression analysis (95% confidence interval) showing relationship between inhibitor-induced reduction in proliferation and driver mutations variant allele-frequence (VAF) in patient samples analyzed in (**A**). Correlations were assessed using two-tailed Pearson’s correlation coefficient. **D** Colony-forming unit (CFU) assays assessing the effects of GLUT inhibition on colony-forming capacity of patient– and HD–derived PBMCs. Cells were seeded with Glutor (1 µM) or KL-11743 (1 µM) at the time of plating. Colonies were quantified after 10–14 days by brightfield microscopy. Colonies numbers were normalized to DMSO (0.02%) controls for each sample and compared across conditions. Statistical analysis was performed using ordinary one-way ANOVA with Tukey’s post-hoc test. All data are presented as mean ± SD. Statistical significance is indicated as follows: *< 0.05, **< 0.01; ***< 0.001, ****< 0.0001

Patient samples (Table S6) were stratified according to the underlying driver mutation (*JAK2*V617F, *CALR*, or *MPL*). Consistent with our previous observations demonstrating selective efficacy of the HIF-1 inhibitor Ech, pharmacological HIF-1 inhibition using ACF significantly reduced both proliferation and viability of PBMCs derived from MPN patients, while effects on PBMCs from HD were markedly weaker (Fig. 5A/B, left) [10].

Glutor significantly reduced proliferation of PBMCs across all driver mutations, while only minimally affecting most HD samples (Fig. 5A/B, middle). Effects on cell viability were less pronounced and mutation-dependent, with significant reductions observed primarily in *CALR– *and *MPL*–mutated samples in comparison to HDs. Co–treatment with the glutaminase inhibitor CB-839 to assess potential redundancy via glutaminase pathways modestly enhanced the anti-proliferative effect, but reduced viability predominantly in *JAK2*V617F–positive samples. Treatment with the GLUT inhibitor KL-11743 yielded results comparable to Glutor (Fig. 5A/B, right). While *JAK2*V617F– and *CALR*–mutated cells exhibited robust reductions in proliferation and viability, *MPL*-mutated cells also demonstrated a trending decrease. This reduction did not achieve statistical significance, likely owing to the limited sample size of this specific subgroup. Co-treatment with CB-839 enhanced the antiproliferative effect of KL-11743 in *MPL*–mutated cells but failed to provide any further reduction in the viability of *JAK2*V617F cells after 7 days of treatment.

Analysis of treatment response relative to mutational burden revealed no negative correlation between Glutor–mediated inhibition of proliferation and driver mutation variant allele frequency (VAF; Fig. 5C, *p* = 0.2422). No such correlation was observed for KL-11743. Notably, ACF treatment displayed a significant negative correlation with driver mutation VAF. This mirrors findings reported for Ech [10], suggesting that VAF-associated sensitivity to HIF-1 inhibition involves mechanisms distinct from the metabolic vulnerabilities targeted by Glutor.

Overall, all tested inhibitors consistently reduced proliferation and, to varying extents, viability of MPN patient–derived cells, while HD–derived cells remained largely unaffected compared with DMSO controls.

To further validate these findings, PBMCs from *JAK2*V617F– and *CALR*–mutated patients were subjected to CFU assays (Fig. 5D). Based on prior demonstration of HIF-1 inhibition effects on JAK2V617F CFU formation [10], these assays focused on Glutor and KL-11743, with CB-839 excluded due to modest effects. The lack of response contrasts with previous reports in *JAK2*V617F patient cells, suggesting context-dependent metabolic differences [39]. Both Glutor and KL-11743 markedly reduced CFU formation in patient–derived samples from both driver mutation groups, while exerting only minor effects on HD–derived colonies (Fig. 5D).

Collectively, these results demonstrate that the metabolic vulnerabilities identified in *Jak2*V617F GLUT1/3 dKO cell lines are partially recapitulated in primary patient material across all common MPN driver mutations using pharmacological inhibition.

## Discussion

In this study, we identify glucose and HIF-1–driven metabolic reprogramming as a central vulnerability in MPN. We demonstrate that oncogenic *JAK2* signaling enforces a hypermetabolic state marked by elevated glycolysis and mitochondrial respiration, specifically dependent on GLUT1 and GLUT3. Pharmacological inhibition with Glutor or genetic ablation of *Slc2a1* and *Slc2a3* effectively abolished glucose uptake and glycolytic flux, with dKO clones exhibiting profound loss of proliferation and viability specifically in the *Jak2*V617F background. The absence of compensatory glucose transport indicates that oncogenic JAK2 does not induce expression of alternative GLUT family members in 32D cells. Residual metabolic activity observed after Glutor treatment was minimal and likely reflects incomplete transporter inhibition or minor glucose entry through non-canonical mechanisms, rather than biologically meaningful compensation [33, 38].

GLUT1 and GLUT3 were specifically selected as targets based on their increased expression in JAK2V617F–positive cells, mirroring observations from solid tumors where overexpression of these transporters is associated with aggressive disease and poor prognosis [40, 41]. This strategy was further supported by the strong efficacy of Glutor, a dual GLUT1/2/3 inhibitor, to effectively abrogate glucose uptake and glycolytic activity in *JAK2*V617F–positive cells. In contrast, no evidence was found for compensatory induction of other glucose transporters upon GLUT1/3 inhibition, indicating that *JAK2*V617F–driven metabolic reprogramming relies predominantly on these transporters rather than broader GLUT family redundancy. Together, these findings justify a focused targeting strategy centered on GLUT1 and GLUT3 as functionally relevant mediators of oncogene–driven glucose addiction.

Consistent with prior reports [14], *Jak2*V617F cells displayed elevated glycolytic flux and overall metabolic activity. Importantly, HIF-1α KD was sufficient to revert this phenotype toward *Jak2*WT–like metabolic states, confirming HIF-1 as a key mediator of *Jak2*V617F–induced metabolic rewiring [10, 42]. While this resembles the classical Warburg effect described in solid tumors [15, 43, 44], our OCR data indicate that *Jak2*V617F cells retain substantial mitochondrial activity even after the metabolic shift towards glycolysis in response to glucose, suggesting a hybrid metabolic state in which glycolysis and oxidative metabolism cooperate to sustain proliferation. This metabolic flexibility has been described by *Fendt et al.* as coordinated gene expression changes in response to fluctuations in nutrient availability as a characteristic feature of primary tumors and is thought to support sustained growth under metabolically constrained conditions [45]. Consistent with this concept, *Kim et al.* reported that glucose metabolism, unlike other nutrient pathways, is recurrently upregulated across diverse oncogenic contexts and hypoxic conditions, while simultaneously representing a shared vulnerability to metabolic disruption, aligning with the classical Warburg framework and its recognition as a hallmark of cancer [46, 47]. In *JAK2*V617F-driven cells, recent work by *Kealy et al.* demonstrated that non-canonical phosphorylation of HIF-1α via PIM1 promotes HIF-1α stabilization and a transcriptional program that is distinct from the classical hypoxia-induced regulon, while still promoting expression of glycolysis-associated genes [48].

The HIF-1–associated increase in glycolytic flux, together with the pronounced apoptosis observed in *Jak2*V617F GLUT dKO cells, provides strong evidence for a state of glucose addiction in this oncogenic context. This concept is supported by *Tata et al.*, who demonstrated that pharmacological inhibition of the glycolytic regulator PFKFB3 using 3PO effectively suppressed growth of human JAK2V617F-positive leukemic cell lines and reversed *Jak2*V617F-induced hypoglycemia in a murine model [14]. Notably, this hypoglycemic phenotype parallels clinical observations by *Mesa et al.*, who reported improvement of cachexia in *JAK*2V617F-positive patients treated with ruxolitinib [49]. While this ruxolitinib-induced reduction in baseline cellular energy demand is beneficial for reversing systemic metabolic wasting, our co-treatment findings reveal a critical, context-dependent dichotomy. Although combining ruxolitinib with Glutor yielded synergistic efficacy in murine 32D *Jak2*V617F cells, it actively antagonized the efficacy of Glutor in human post-MPN AML cell lines (SET-2 and HEL) by inadvertently shielding the cells from acute starvation. This highlights the necessity of carefully selecting co-treatment partners based on the specific metabolic landscape of the disease model.

Beyond its role in adenosine triphosphate (ATP) generation, glucose uptake is essential for sustaining pentose phosphate pathway flux, amino acid and nucleotide biosynthesis, and cellular redox homeostasis. Accordingly, *Kang et al.* showed that glucose-addicted cancer cells rely on glucose metabolism to maintain reactive oxygen species (ROS) balance, with glucose deprivation inducing apoptosis primarily through redox stress rather than energy depletion [50]. In contrast, *Rodriguez et al.* identified a selective vulnerability of leukemic stem cells to GLUT1 inhibition in murine AML models, where impaired glucose uptake triggered autophagy and cell death due to loss of bioenergetic homeostasis [20]. These effects can provide a mechanistic framework for the selective vulnerability of *Jak2*V617F–mutated cells to perturbations in glucose metabolism and are consistent with previous reports demonstrating the therapeutic potential of targeting glycolytic regulators in MPN [14].

Transcriptomic profiling further underscores the extent to which *JAK2*V617F–driven cells rely on GLUT1/3–mediated glucose uptake to sustain metabolic homeostasis and proliferation. Loss of GLUT1/3 dysregulated not only HIF-1 signaling, glycolysis, and amino acid metabolism, but also key processes of cell cycle regulation, DNA replication, and DNA repair. Rather than inducing compensatory metabolic rewiring, these changes culminated in a transcriptional signature characteristic of metabolic collapse. The pronounced induction of stress– and apoptosis–associated genes, validated by RT-qPCR, underscores the inability of *Jak2*V617F dKO cells to adapt to glucose deprivation, ultimately triggering cell cycle failure during S-phase and cell death, while *Jak2*WT cells remain able to progress towards late S-phase.

In contrast, *Jak2*V617F GLUT WT clones exhibited a markedly milder transcriptional stress response compared to *Jak2*WT cells, while maintaining OXPHOS and amino acid metabolism, contrasting their elevated rates of basal apoptosis. However, this discrepancy suggests that *Jak2*V617F cells, already burdened by the intrinsic stress of the oncogenic state, adopt a protective adaptation under moderate nutrient limitation by actively repressing transcriptional death pathways, as indicated by its transcriptional signature [51–53]. This is further validated by increased populations of *Jak2*V617F cells found in a resting G0/G1 phase in nutrient-poor conditions.

These findings reinforce the concept that *JAK2*V617F confers a heightened metabolic dependency on glucose transport, as the lack of effective compensatory adaptation in dKO cells further validates glucose transport as a selective and exploitable therapeutic target in JAK2V617F–positive MPN. We propose that, in line with our MTT and proliferation assay findings, heightened glucose dependency driven by *Jak2*V617F induces a protective state under glucose-limited culture conditions, suppressing proliferation and metabolic demand to maximize nutrient uptake and preserve viability. In contrast, complete loss of glucose transport in dKO cells precipitated terminal stress and cell death.

Despite these compelling in vitro findings, pharmacological targeting of HIF-1 or glucose transporters failed to robustly ameliorate disease features in a murine *Jak2*V617F model. Rather than contradicting our hypothesis, these negative in vivo results highlight key translational challenges: short plasma half–life, suboptimal bioavailability, and limited tissue penetration of the compounds used likely constrained target engagement [54, 55]. Evidence of altered erythroid precursor distribution, with reduced BM erythropoiesis accompanied by increased splenic erythropoiesis, suggests partial on-target effects that may be masked by compensatory extramedullary hematopoiesis. Such niche–dependent resistance mechanisms are well described in MPN and may protect malignant HSPCs outside the BM microenvironment [56]. These observations suggest that the drugs likely engaged their intended metabolic targets, but the disease adapts spatially rather than molecularly, shifting erythropoiesis to extramedullary sites such as the spleen, which provides a protective microenvironment [57]. A further limitation of the in vivo study is the absence of a positive control treatment known to attenuate the MPN phenotype in this model (e.g., a JAK inhibitor). Inclusion of such a benchmark could have helped validate model responsiveness and contextualize the lack of therapeutic efficacy observed with metabolic inhibition. These limitations are nonetheless informative. They indicate that metabolic targeting may require optimized drug formulations, altered dosing schedules, or combination strategies to overcome pharmacokinetic constraints and microenvironmental protection. In this context, combining metabolic inhibitors with agents that reduce splenic hematopoiesis or disrupt niche–mediated protection may represent a rational therapeutic strategy [58–60]. Furthermore, future in vivo competitive transplantation studies will be necessary to formally evaluate the impact of GLUT inhibition on mutant clonal fitness and the competitive dynamics between JAK2V617F and JAK2WT cells.

Beyond the pharmacological challenges observed in vivo, several limitations warrant consideration. 32D cells, while providing a clean genetic system for mechanistic dissection of functional redundancy between GLUT1 and GLUT3, are an immortalized murine cell line and may not perfectly capture the metabolic nuances of primary HSPCs. Nevertheless, our ability to successfully confirm the efficacy of Glutor in primary patient-derived blood cells and human JAK2V617F-mutated cell lines (HEL and SET-2) firmly underscores the translational relevance of these metabolic vulnerabilities. Furthermore, standard in vitro culture conditions do not replicate the complex, nutrient-restricted and hypoxic bone marrow microenvironment. This environmental gap may mask the metabolic plasticity of malignant clones. While we maintained dKO clones for several weeks without observing the emergence of compensatory mechanisms, the potential for acquired resistance through the long-term upregulation of non-canonical transport or bypass signaling remains a possibility in the more selective in vivo environment, necessitating further longitudinal study. In a living system, these cells might pivot to alternative substrates to bypass a compromised glucose transport system.

Crucially, our findings translate to primary patient material. In vitro treatment of patient–derived PBMCs with HIF-1 and GLUT inhibitors selectively reduced cell viability and exerted antiproliferative effects across *JAK2*V617F–, *CALR*–, and *MPL*–mutated samples, while sparing unmutated controls. While this observation hints that HIF-1–dependent glucose metabolism might represent a shared therapeutic vulnerability across genetically distinct MPN subtypes, our current mechanistic data are confined to the JAK2V617F setting. To determine whether CALR- and MPL-mutated cells undergo identical metabolic rewiring, extensive metabolic characterization in dedicated, driver-specific in vitro models will be required in future studies.

Additionally, while our results in primary patient samples were encouraging, the inherent clonal heterogeneity of MPN suggests that rare sub-clones or co-occurring mutations (e.g., TET2, ASXL1) might possess distinct metabolic signatures that provide resistance to GLUT1/3 inhibition.

## Conclusions

Taken together, our data define glucose uptake and HIF-1–dependent metabolic reprogramming as fundamental features of MPN biology that can be selectively targeted in vitro and in patient–derived cells. While in vivo efficacy remains to be established, the observed niche–dependent responses and pharmacological limitations provide a clear roadmap for rational optimization. With improved drug delivery and combination strategies, metabolic targeting may offer a complementary approach to selectively impair malignant hematopoiesis in MPN.

## Supplementary Information

Supplementary Material 1.

## Data Availability

The RNAseq data generated in this study have been deposited in the Gene Expression Omnibus (GEO) under accession number GSE319980. All other data supporting the findings of this study are available from the corresponding author upon reasonable request.

## Abbreviations

AA: Amino acid
ACF: Acriflavine
AnnV: Annexin V
ATP: Adenosine triphosphate
BM: Bone marrow
BrdU: 5’-bromo-2’deoxyuridine
CFU: Colony-forming units
dKO: Double KO
ECAR: Extracellular acidification rate
Ech: Echinomycin
ET: Essential thrombocythemia
EV: Empty vector
FA: Fatty acid
FCS: Fetal calf serum
gRNA: GuideRNA
HD: Healthy donor
HCT: Hematocrit
HGB: Hemoglobin
HIF-1: Hypoxia-inducible factor 1
HRE: Hypoxia-response elements
HSC: Hematopoietic stem cell
i.p.: Intraperitoneal
IL-3: Interleukin-3
KD: Knockdown
KI: Knock-in
KO: Knockout
MPN: Myeloproliferative neoplasm
OCR: Oxygen consumption rate
OXPHOS: Oxidative phosphorylation
PB: Peripheral blood
PBMC: Peripheral blood mononuclear cell
PBS: Phosphate-buffered saline
PMF: Primary myelofibrosis
PV: Polycythemia vera
RBC: Red blood cell
ROS: Reactive oxygen species
RNAseq: RNA sequencing
RT-qPCR: Reverse transcriptase quantitative PCR
Scr: Scrambled controls
WT: Wild-type
VAF: Variant allele frequency
