# Supplementary material for: Glucose transport dependency defines a therapeutic vulnerability in JAK2V617F-driven myeloproliferative neoplasms
Source: Cell Commun Signal. 2026 Jun 23;24:370. doi: 10.1186/s12964-026-03018-4 (PMC13288585; doi:10.1186/s12964-026-03018-4)
Supplement: Supplementary file 1 — Supplementary Material 1. [file 12964_2026_3018_MOESM1_ESM.docx]

**Glucose Transport Dependency Defines a Therapeutic Vulnerability in JAK2V617F-driven Myeloproliferative Neoplasms**

**Authors**

Patrick Weiand^1,2^, Nicolas Chatain^1,2^, Marcelo A. Szymanski de Toledo^1,2^, Julia Moellmann^3^, Tabea Pirker^3^, Siddharth Gupta^1,2^, Henrike Jacobi^1,2^, Jelena Lazarevic^1,2^, Margherita Vieri^1,2^, Maria Jimena Rodriguez^1,2^, Steffen Koschmieder^1,2^, Deniz Nogueira Gezer^1,2,*^ & Julian Baumeister^1,2,*,✉^

^1^ Department of Hematology, Oncology, Hemostaseology, and Stem Cell Transplantation, Faculty of Medicine, RWTH Aachen University, Aachen, Germany

^2^ Center for Integrated Oncology Aachen Bonn Cologne Düsseldorf (CIO ABCD), Aachen, Germany

^3^ Department of Internal Medicine I, Cardiology, University Hospital RWTH Aachen, Aachen, Germany

^*^ Contributed equally
^✉^ Corresponding author (jbaumeister@ukaachen.de)

**Supplementary Information**

**1 Supplementary Tables**

**Table S1: guideRNA sequences targeting GLUT1 and GLUT3**

| **Target** | **Name** | **Strand** | **Sequence** | **PAM** |
| --- | --- | --- | --- | --- |
| *Slc2a1* Exon 3 | Mm.Cas9.SLC2A1.1.AD | + | CAAACATGGAACCACCGCTA | CGG |
| *Slc2a1* Exon 4 | Mm.Cas9.SLC2A1.1.AE | + | GCTTATGGGCTTCTCCAAAC | TGG |
| *Slc2a3* Exon 5 | Mm.Cas9.SLC2A3.1.AD | - | GGACTCTTTGTCAACCGCTT | TGG |

gRNA sequences were obtained from the Integrated DNA Technology (IDT, Coralville, USA) library for pre-designed AltR CRISPR-Cas9, and subsequently also purchased from IDT).

**Table S2: Primers used in CRISPR screening**

| **Target** | **Sequences** |
| --- | --- |
| *Slc2a1* E3-E4 fw | CCCAGTCCTCTTTTCGTCCG |
| *Slc2a1* E3-E4 rv | CCTGGGCAATAAGGATGCCA |
| *Slc2a1* AD off-target fw | AGAAGGTCATTTCAGGCACGG |
| *Slc2a1* AD off-target rv | TTGGCGTGTTATGCATATGTCC |
| *Slc2a1* AE off-target fw | GCCTAGATGTCTCCAATCTTCCAA |
| *Slc2a1* AE off-target rv | GGTATCCTGTGCACAAACAGAG |
| *Slc2a3* Exon 5 fw | AAGGAACTCCGTTGCTGACC |
| *Slc2a3* Exon 5 rv | AGCCACCCTAGGATTTGCCT |
| *Slc2a3* AD off-target fw | GCAGTGAGCAGAGGAGTACC |
| *Slc2a3* AD off-target rv | ACCTCTCTCCTGTTGACCTAC |

Primers were designed using Primer-BLAST from the U.S. National Library of Medicine (<https://www.ncbi.nlm.nih.gov/tools/primer-blast/index.cgi>). Selected Primers were ordered from MWG Eurofins.

**Table S3: Primers used in RT-qPCR analyses**

| **Target** | **Sequences** |
| --- | --- |
| *Hprt* fw | GGGGGCTATAAGTTCTTTGC |
| *Hprt* rv | TCCAACACTTCGAGAGGTCC |
| *Chek1** fw | GTTAAGCCACGAGAATGTAGTGA |
| *Chek1** rv | GATACTGGATATGGCCTTCCCT |
| *Pcna** fw | TTTGAGGCACGCCTGATCC |
| *Pcna** rv | GGAGACGTGAGACGAGTCCAT |
| *E2f1** fw | TGCAGAAACGGCGCATCTAT |
| *E2f1** rv | CCGCTTACCAATCCCCACC |
| *Pidd1* fw | TCCTTGTTCTGCACAGCAACCT |
| *Pidd1* rv | AACCTGGGATATGTCTGGGGGA |

Primers were designed using Primer-BLAST from the U.S. National Library of Medicine (<https://www.ncbi.nlm.nih.gov/tools/primer-blast/index.cgi>). *-marked Primers were obtained from the Harvard PrimerBank of the Harvard Medical school database for quantitative gene expression analysis PCR primers (<https://pga.mgh.harvard.edu/primerbank/index.html>). Selected Primers were ordered from MWG Eurofins.

**Table S4: Antibodies**

| **Antibody** | **Use** | **Company** |
| --- | --- | --- |
| Ter119-APC | Flow cytometry | Biolegend, USA |
| CD71-PE | Flow cytometry | Biolegend, USA |
| GLUT1 Recombinant Rabbit Monoclonal Antibody (SA0377) | Western blot | Thermo Fisher Scientific, USA |
| GLUT3 Recombinant Rabbit Polyclonal Antibody (PA5-99486) | Western blot | Thermo Fisher Scientific, USA |
| GAPDH mouse monoclonal antibody (6C5) | Western blot | Santa Cruz Biotechnology, USA |
| Pacific Blue Annexin V | Flow cytometry | Biolegend, USA |

**Table S5: Gene edits in KO clones**

| **32D MPL^+/+^** | | | | | | | | | | | | |
| --- | --- | --- | --- | --- | --- | --- | --- | --- | --- | --- | --- | --- |
| ***Jak2*WT** | | | | | | | | | | | | |
| Group | GLUT1/3 WT | | | GLUT1 KO | | | GLUT3 KO | | | GLUT1/3 dKO | | |
| Clone Nr. | **1** | **2** | **3** | **4** | **5** | **6** | **7** | **8** | **9** | **10** | **11** | **12** |
| Indel mutations (bp) **GLUT1** | **/** | **/** | **/** | **-262** | **-1 / -17** | **-262** | **/** | **/** | **/** | **-262** | **-262** | **-262** |
| Indel mutations (bp) **GLUT3** | **/** | **/** | **/** | **/** | **/** | **/** | **-1** | **-8** | **-35** | **-1** | **-5** | **-1** |
| ***Jak2*V617F** | | | | | | | | | | | | |
| Group | GLUT1/3 WT | | | GLUT1 KO | | | GLUT3 KO | | | GLUT1/3 dKO | | |
| Clone Nr. | **1** | **2** | **3** | **4** | **5** | **6** | **7** | **8** | **9** | **10** | **11** | **12** |
| Indel mutations (bp) **GLUT1** | **/** | **/** | **/** | **-347** | **-129 / -214** | **-347** | **/** | **/** | **/** | **-347** | **-347** | **-347** |
| Indel mutations (bp) **GLUT3** | **/** | **/** | **/** | **/** | **/** | **/** | **-3 / -7** | **-2 / +18** | **-3 / -8** | **-1 / -12** | **-12** | **-2 / -37** |

**Table S6: Patient material information**

| **Patient Nr.** | **Diagnosis** | **Driver-Mutations** | **VAF %** | **Co-Mutations** |
| --- | --- | --- | --- | --- |
| 1 | Post-PV-MF | JAK2 V617F | 85 | TET2 N1387T (5,7%) |
| 2 | PV | JAK2 V617F | 59 | TET2 Q916* (29%) |
| 3 | Post-PV-MF | JAK2 V617F | 78 | - |
| 4 | Post-ET-MF | JAK2 V617F | 48 | - |
| 5 | PV | JAK2 V617F | 78 | TET2 Q876* (40%) |
| 6 | PV | JAK2 V617F | 14 | - |
| 7 | PV | JAK2 V617F | 57 | TET2 2-Bp Del p.His974Glnfs*8 (32%) |
| 8 | PV | JAK2 V617F | 40 | - |
| 9 | PV | JAK2 V617F | 28 | - |
| 10 | ET | CALR (5 Bp Ins) | 32 | - |
| 11 | PV | JAK2 V617F | 50 | - |
| 12 | PV | JAK2 V617F | 45 | TET2 Q325* (5,3%) E1352K (6,6%) |
| 13 | PV | JAK2 V617F | 29 | - |
| 14 | PMF | CALR (52 Bp del) | 51 | ASXL1 Q588* (8,2%) |
| 15 | PV | JAK2 V617F | 23 | - |
| 16 | ET | CALR (19 Bp del) | 44 | - |
| 17 | ET | CALR (52 Bp del) | 30 | - |
| 18 | ET | MPL W515K | 46 | - |
| 19 | PV | JAK2 V617F | 53 | - |
| 20 | PV | JAK2 V617F | 76 | EZH2 delins p.Phe632Leufs*43 (8,4%) |
| 21 | PV | JAK2 V617F | 97 | TET2 T1397I (32%) |
| 22 | PMF | JAK2 V617F | 24 | - |
| 23 | PV | JAK2 V617F | 3,2 | - |
| 24 | ET | JAK2 V617F | 40 | - |
| 25 | PMF | JAK2 V617F | 92 | - |
| 26 | PV | JAK2 V617F | 80 | - |
| 27 | ET | MPL W515L | 40 | - |
| 28 | PMF | JAK2 V617F | 34 | - |
| 29 | PMF | CALR (52 Bp del) | 57 | - |
| 30 | PV | JAK2 V617F | 34 | ASXL1 E5931* (18%) |
| 31 | präPMF | CALR (52 Bp del) | 49 | - |
| 32 | präPMF | CALR (52 Bp del) | 53 | - |
| 33 | ET | CALR (46 Bp del) | 28 | - |
| 34 | PMF | JAK2 V617F | 82 | - |
| 35 | Post-ET-MF | MPL S204P | 84 | - |
| 36 | ET | CALR (5 Bp ins) | 44 | - |
| 37 | ET | CALR (52 Bp del) | 52 | ASXL1 1 Bp-Dup p.Tjr118Asnsfs*9 (27%) TET2 1 Bp-del p.Ser271Glnfs*22 (28%) TET2 1 Bp-del p.Gln273Argfs*20 (28%) - same allel |
| 38 | PV | JAK2 V617F | 52 | ASXL1 1 bp-Dup p.Ser892Phefs*2 (18%) |
| 39 | Post-ET-MF | JAK2 V617F | 69 | - |
| 40 | PV | JAK2 V617F | 22 | - |
| 41 | präPMF | CALR (31 Bp del) | 42 | - |
| 42 | PMF | CALR (5 Bp del) | 53 | - |
| 43 | ET | CALR (52 Bp del) | 49 | - |
| 44 | präPMF | MPL W515L | 41 | - |
| 45 | PMF | MPL W515L | 69 | MPL R592Q (70%) |
| 46 | PMF | CALR (5 Bp ins) | 36 | - |

Screening for additional mutations in ASXL1, CBL, CHECK2, DNMT3A, IDH2, KRAS, SETBP1, SRSF2, TET2 and U2AF1 was performed with patient PBMCs using next generation sequencing. Allel burdens are indicated in brackets. No additional mutations present are denoted as -.

**2 Supplementary Figures**


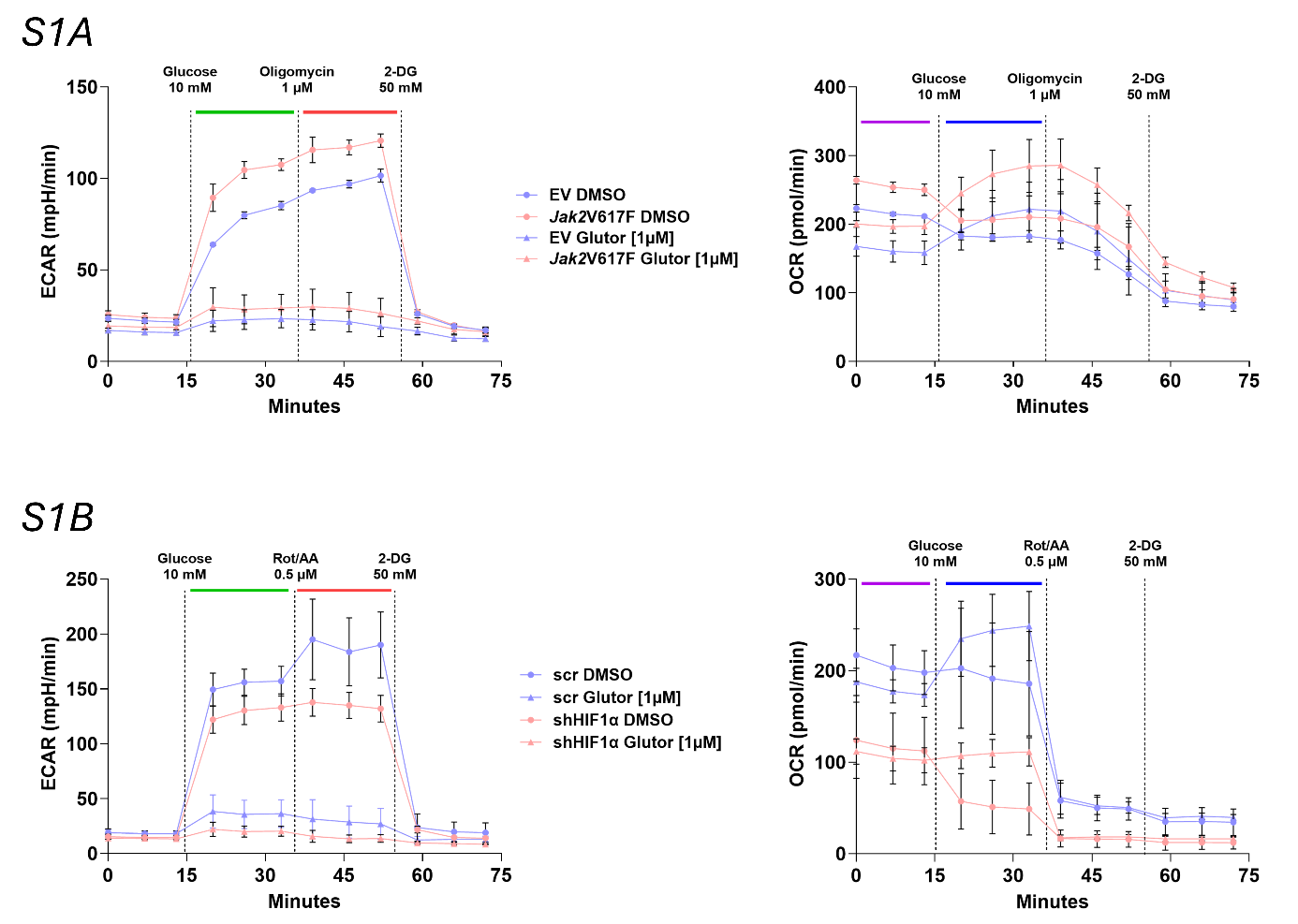


**Fig S1. Full extracellular flux time courses underlying Fig. 1:** **(A)** Full time course of extracellular flux measurements, separated into ECAR (left) and OCR (right), in 32D EV and *Jak2*V617F cells treated with either DMSO (0.01%) or Glutor (1 µM). **(B)** Full time course of extracellular flux measurements, separated into ECAR (left) and OCR (right), in 32D *Jak2*V617F cells expressing small hairpin-RNA (shRNA) targeting HIF-1α (sh*Hif1a)* or scrambled control (scr) treated with either DMSO (0.01%) or Glutor (1 µM). In both panels, ECAR and OCR were measured at three subsequent timepoints in each phase at 3 min intervals. Compounds indicated above curves were injected into each well at indicated concentrations and timepoints. Shaded regions correspond to the time windows used for quantification and data shown in Fig. 1A-C (A) or Fig. 1D-F (B). Data are presented as mean ± SD of three independent experiments (n=3), with 8 technical replicates averaged for each condition at each timepoint per n.


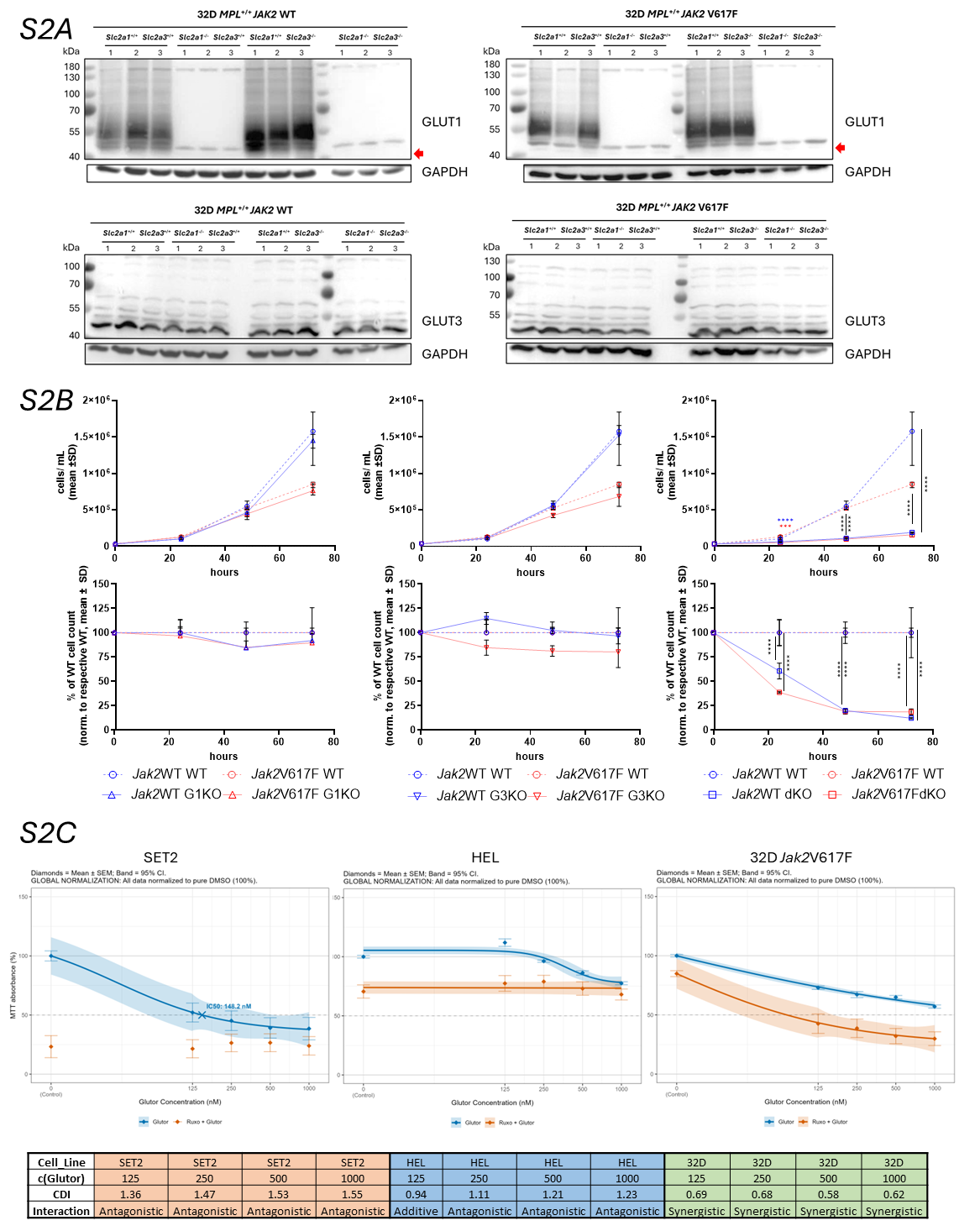


**Fig S2. GLUT1 and GLUT3 protein expression, proliferation of knockout clones, and MTT assays with SET, HEL, and 32D cells: (A)** Representative Western blot analysis of GLUT1 (top) or GLUT3 (bottom) protein expression in 32D *MPL*WT *Jak2*V617F *Slc2a1* (GLUT1) or *Slc2a3* (GLUT3) wild-type (WT), single-knockout or double-knockout 32D cells with GAPDH as loading control (n=3). Total of 20 µg of protein lysates were loaded into each pocket per samples. Antibody details are provided in Table S4. **(B)** Proliferation of GLUT1 or GLUT3 KO clones was assessed over 72 h. 1 x 10^5^ cells were seeded in low-supplement RPMI-1640, and cell counts were measured every 24 h using a CASY cell counter. Data are presented as absolute cell numbers per mL of culture medium (top) or normalized to the respective GLUT1 or GLUT3 WT clones (bottom). Data are presented as mean ± SD from three independent experiments (n=3), with three independent clones used as replicates per condition for each experiment. Statistical analysis was performed using ordinary two-way ANOVA and Tukey’s multiple comparison test. **(C)** MTT assays were performed with JAK2V617F-mutated cell lines (HEL, SET-2, and 32D MPL *Jak2*V617F) following 72 hours of treatment with varying concentrations of Glutor, either alone or in combination with 500 nM ruxolitinib. Relative metabolic activity was determined by measuring absorbance at 550 nm and is normalized to vehicle (DMSO) controls. The Coefficient of Drug Interaction (CDI) was calculated to evaluate the combination effect, where a CDI < 0.9 indicates synergy, 0.9–1.1 indicates additivity, and > 1.1 indicates antagonism.


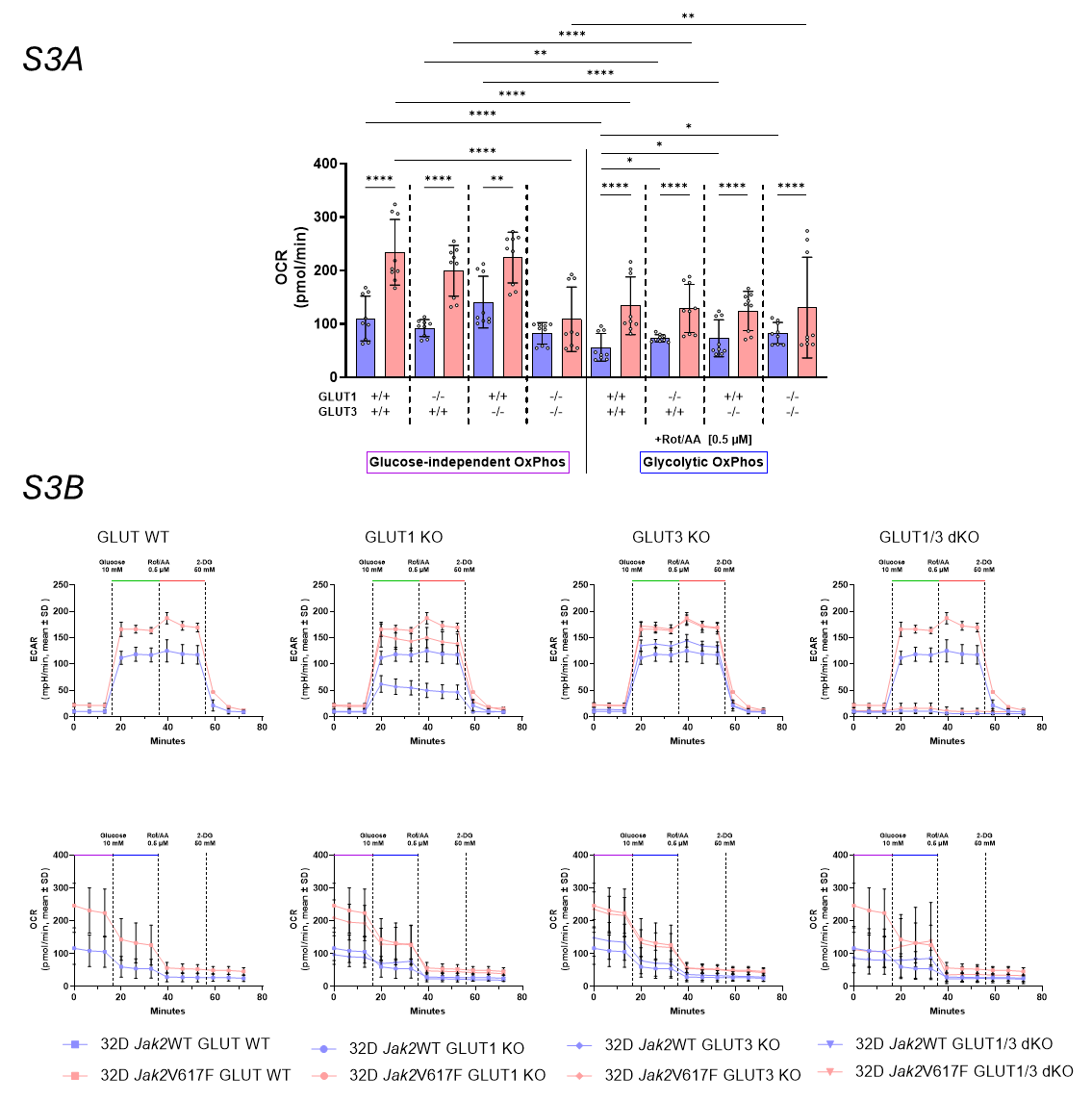


**Fig S3. Extracellular flux profiles of GLUT knockout clones: (A)** OCR changes of all 32D GLUT wild-type (WT) and knockout (KO) clones in both genetic backgrounds before (left) and after glucose addition (right; 10 mM). Statistical analysis was performed using ordinary one-way ANOVA with Šídák multiple comparison tests within and between metabolic phases. Each clone per condition was measured once per independent experiment (n=3, 3 measured timepoints per phase per n, each timepoint averaged from 8 analytical replicates per condition). **(B)** Full time course of extracellular flux measurements, separated into ECAR (top) and OCR (bottom), of 32D *Jak2*WT and *Jak2*V617F KO clones. Flux was measured at three subsequent timepoints in each phase at 3 min intervals. Compounds indicated above curves were injected into each well at indicated concentrations and timepoints. Shaded regions correspond to the time windows used for quantification and data shown in Fig. 2C (top) or Fig. S3A (bottom). Data are presented as mean ± SD of three independent experiments (n=3), with 8 technical replicates averaged for each condition at each timepoint per n.


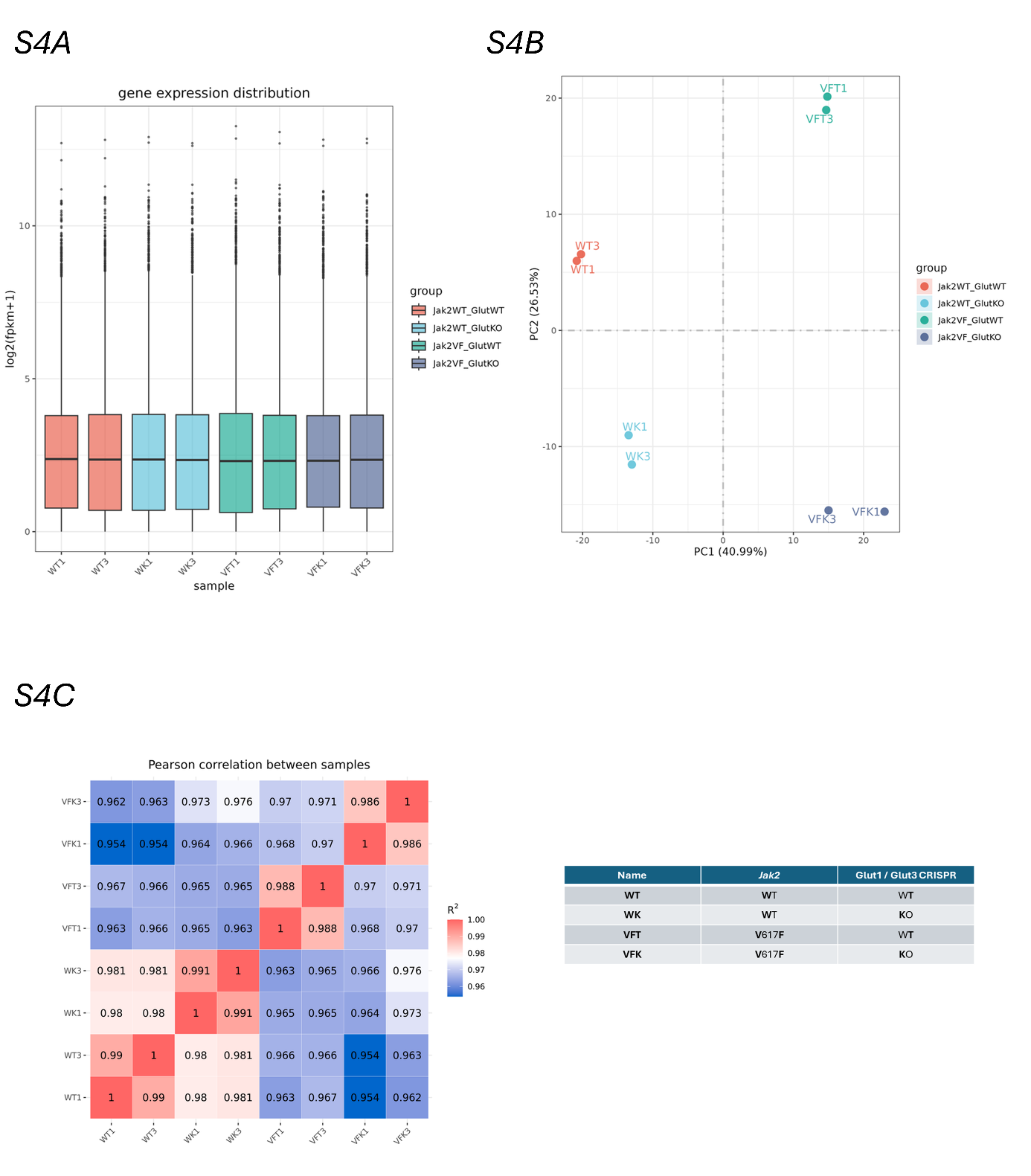


**Fig S4. Global quality assessment and clustering of RNA-seq dataset: (A)** Distribution of gene expression values across all RNA-seq samples, including two GLUT1/GLUT3 wild-type (GlutWT) and double-knockout (GlutKO) clones of *Jak2*WT and *Jak2*V617F genetic background, respectively, shown as log_2_(FPKM+1), illustrating overall expression ranges and comparability between sequencing libraries. **(B)** Principal component analysis (PCA) of all RNA-seq samples summarizing variances between samples and clutersting according to genetic background and clone. The table below the plot indicates the assignment of samples to genetic groups. **(C)** Pearson correlation matrix of all RNA-seq samples displayed as heatmap, illustrating similarities (red) and dissimilarities (blue) in global gene expression patterns.


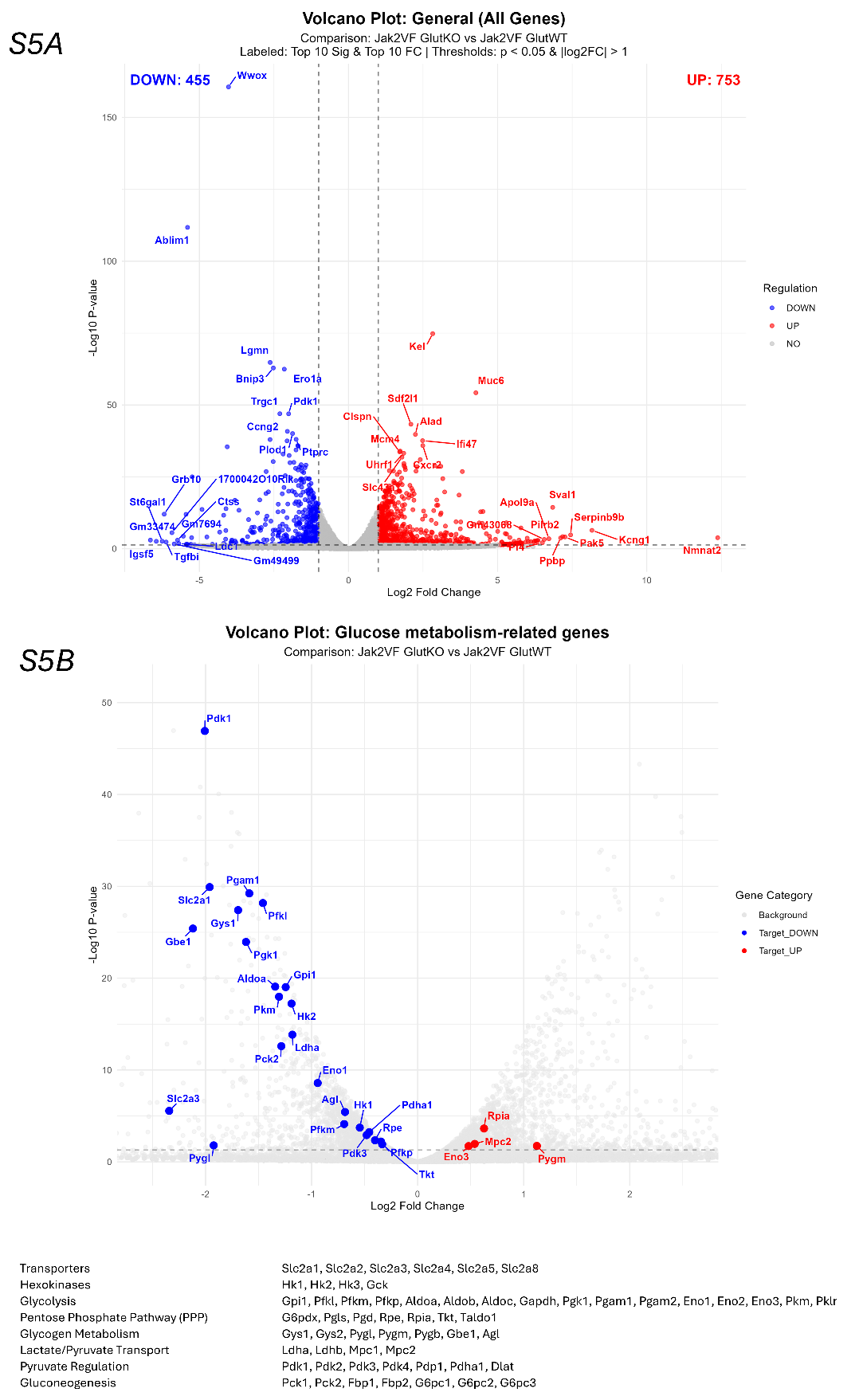


**Fig S5. Differential expression and pathway-focused transcriptional analysis of *Jak2*V617F GLUT1/GLUT3 double-knockout versus wild-type cells: (A)** Volcano plot displaying differentially expressed genes identified by RNA-seq in the comparison of GLUT1/GLUT3 double-knockout (GlutKO) vs wild-type (GlutWT) *Jak2*V617F cells. Genes with an absolute log₂ fold change ≥ 1 and p ≤ 0.05 are highlighted, with downregulated genes shown in blue and upregulated genes in red. **(B)** Volcano plot of the same dataset as in (A)., Selected genes based on their role in glucose metabolism-related pathways are highlighted (p≤ 0.05) to visualize pathway-specific transcriptional changes. A list of targeted genes and their corresponding pathways or families is shown below the plot.


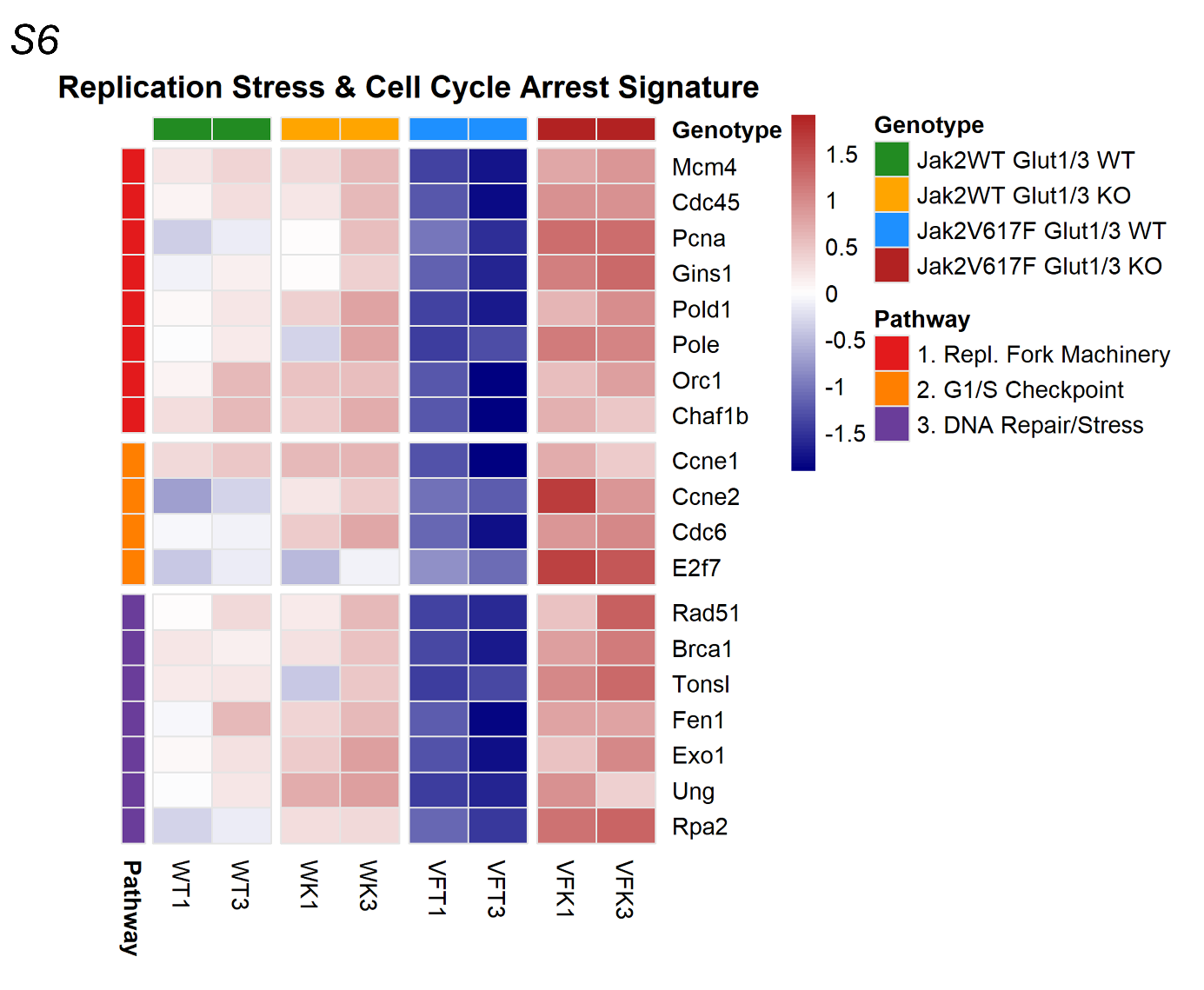


**Fig S6. *Jak2*V617F GLUT1/3 wild-type cells display decreased replication signatures in response to nutrient stress:** Heatmap showing normalized expression of key genes from curated replication stress and cell cycle gene sets across indicated groups. Rows correspond to individual genes, clustered according to their gene sets. Gene expression values are scaled per gene, with higher expression depicted in red and lower in blue.


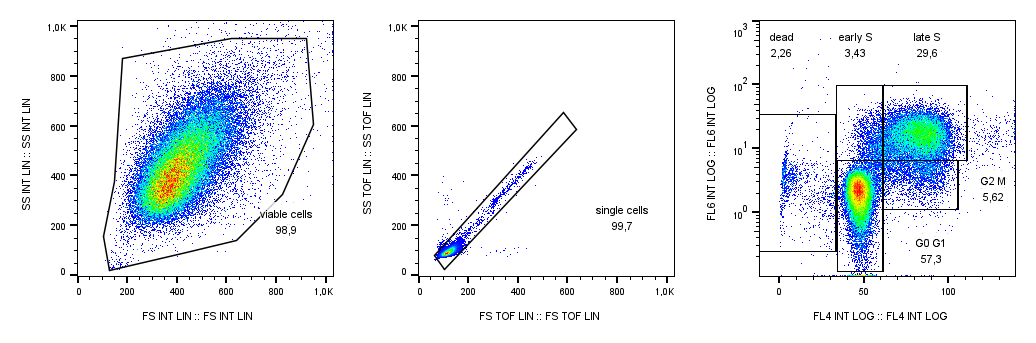


**Fig S7. Gating strategy for BrdU/7-AAD flow cytometry analysis: (A)** Gating strategy utilized to identify Sub G1, G0/G1, early S, late S and G2/M phase populations based on BrdU and 7-AAD stainings in 32D *Jak2*WT and *Jak2*V617F GLUT1/3 WT and dKO cells, as analyzed in Fig 4C.


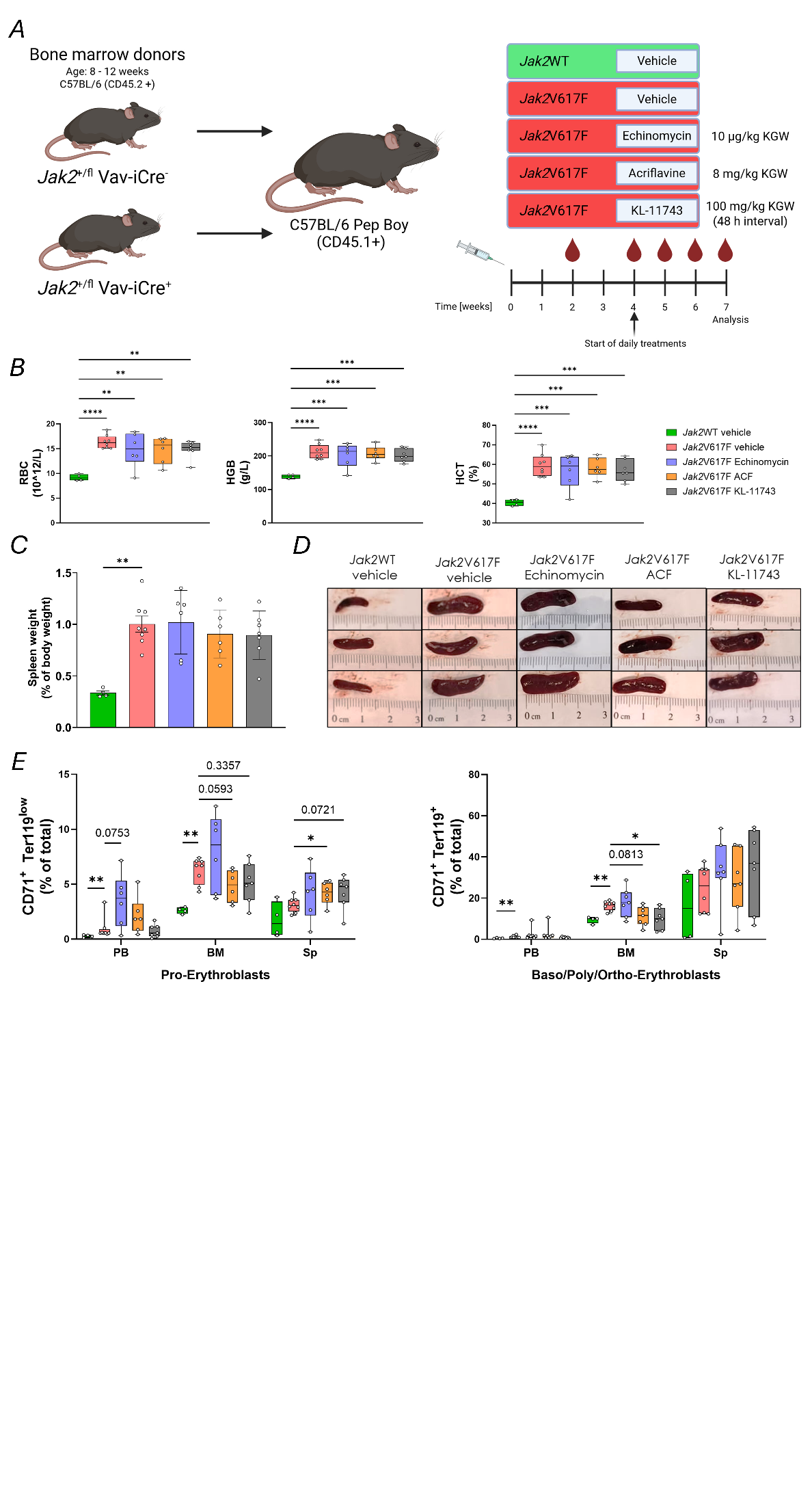


**Fig S8. Limited in vivo effects of HIF-1 and GLUT inhibition in a *Jak2*V617F knock-in model. (A)** Schematic overview of the experimental design, including mouse strains, irradiation, transplantation, experimental timeline, blood collection timepoints, treatment groups, dosages, and administration schedules. **(B)** Hemograms showing red blood cell (RBC; left) counts and hemoglobin (HGB; right) levels in each experimental group before treatment initiation (week 4) versus finalization (week 7). Statistical analysis was performed using ordinary One-way ANOVA with Dunnett’s post-hoc test. **(C)** Spleen weights at study finalization, normalized to individual body weight. All groups were compared to the *Jak2*WT vehicle group using Kruskal-Wallis testing with Dunn’s post-hoc correction. **(D)** Representative images of spleens from each experimental group after extraction, placed next to a scale bar. **(E)** Frequencies of erythroid progenitor populations in peripheral blood (PB), bone marrow (BM), and spleen (Sp), identified by flow cytometry following anti-CD71 and anti-Ter119 staining. Pro-erythroblasts (left) and basophilic/polychromatic/orthochromatic erythroblasts (right) are shown as percentages of total measured cells. Data were analyzed using pairwise comparisons between *Jak2*V617F vehicle–treated mice with each treatment group using Mann-Whitney test. All data are presented as mean ± SD. Statistical significance is indicated as follows: ***< 0.05, **< 0.01; ***< 0.001, ****< 0.0001.

Between weeks 4 and 7, all Jak2V617F mice developed a PV–like phenotype characterized by splenomegaly and increased red blood cell (RBC) counts, hemoglobin (HGB) and hematocrit (HCT) in peripheral blood (PB, Fig. 5B-D). None of the administered treatments resulted in a significant reduction of spleen weight or normalization of RBC, HGB or HCT values, indicating that the disease was not effectively attenuated under the applied treatment conditions. Flow cytometric analysis of PB, BM, and spleen revealed modest treatment–associated changes in erythroblast populations during nucleated stages of erythropoiesis (Fig. S8E, gating strategy shown in Fig. S9). As expected, all Jak2V617F groups exhibited an expansion of erythroblast populations compared with controls, consistent with the observed hematologic phenotype


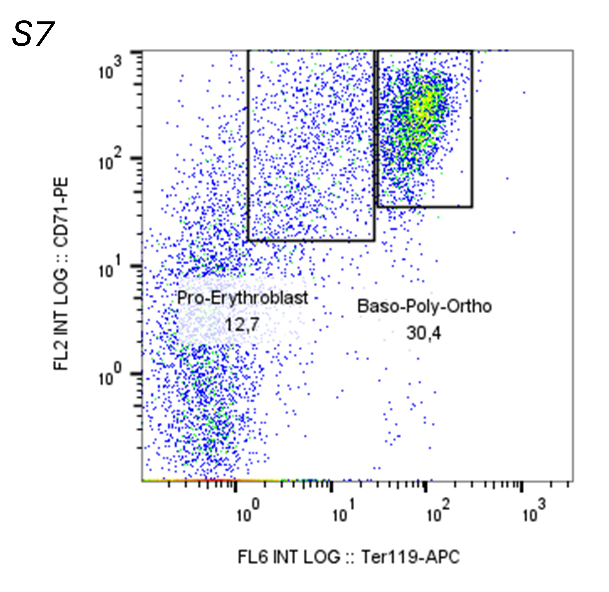


**Fig S9. Gating strategy for CD71/Ter119 flow cytometry analysis: (A)** Gating strategy utilized to identify erythroblast populations based on CD71 and Ter119 cell surface expression in peripheral blood, bone marrow and spleen samples, as analyzed in Fig. S8E.
